# Random regression for modeling yield genetic trajectories in *Jatropha curcas* breeding

**DOI:** 10.1371/journal.pone.0244021

**Published:** 2020-12-23

**Authors:** Marco Antônio Peixoto, Rodrigo Silva Alves, Igor Ferreira Coelho, Jeniffer Santana Pinto Coelho Evangelista, Marcos Deon Vilela de Resende, João Romero do Amaral Santos de Carvalho Rocha, Fabyano Fonseca e Silva, Bruno Gâlveas Laviola, Leonardo Lopes Bhering

**Affiliations:** 1 Universidade Federal de Viçosa, Viçosa, MG, Brazil; 2 INCT Café/Universidade Federal de Viçosa, Viçosa, MG, Brazil; 3 Embrapa Café/Universidade Federal de Viçosa, Viçosa, MG, Brazil; 4 Embrapa Agroenergia, Brasília, DF, Brazil; Institute of Mediterranean Forest Ecosystems of Athens, GREECE

## Abstract

Random regression models (RRM) are a powerful tool to evaluate genotypic plasticity over time. However, to date, RRM remains unexplored for the analysis of repeated measures in *Jatropha curcas* breeding. Thus, the present work aimed to apply the random regression technique and study its possibilities for the analysis of repeated measures in *Jatropha curcas* breeding. To this end, the grain yield (GY) trait of 730 individuals of 73 half-sib families was evaluated over six years. Variance components were estimated by restricted maximum likelihood, genetic values were predicted by best linear unbiased prediction and RRM were fitted through Legendre polynomials. The best RRM was selected by Bayesian information criterion. According to the likelihood ratio test, there was genetic variability among the *Jatropha curcas* progenies; also, the plot and permanent environmental effects were statistically significant. The variance components and heritability estimates increased over time. Non-uniform trajectories were estimated for each progeny throughout the measures, and the area under the trajectories distinguished the progenies with higher performance. High accuracies were found for GY in all harvests, which indicates the high reliability of the results. Moderate to strong genetic correlation was observed across pairs of harvests. The genetic trajectories indicated the existence of genotype × measurement interaction, once the trajectories crossed, which implies a different ranking in each year. Our results suggest that RRM can be efficiently applied for genetic selection in *Jatropha curcas* breeding programs.

## Introduction

*Jatropha curcas* L. ranks among the most relevant crops for biofuel production [1,2]. Its high adaptability, which allows its cultivation under different environmental conditions, tolerance to drought, longevity and high oil quality are some desirable characteristics which highlight *Jatropha curcas* as an excellent alternative for renewable energy production [1,3,4].

It has a high oil content in its seeds (up to 35%), with a high oil-to-biofuel conversion efficiency, compared to other species [4,5]. After extracted, the oil is composed by 47% crude fat and 25% crude protein; and, when compared to other vegetable oils, it presents better oxidation stability and lower viscosity [6].

*Jatropha curcas* is a perennial plant that produces during several years, and in its breeding process, genetic selection has been carried out considering only one harvest [7], several harvests independently [8] or several harvests simultaneously [9,10].

It is worth to highlight some specific issues in perennial plant breeding that affect genetic selection. Once repeated measures are taken in the same individuals over time, analyses must consider: the permanent environmental effects and genetic and residual covariance among harvests. Thus, for quantitative traits, genetic selection must consider several harvests to maximize the selective accuracy [11].

In this context, random regression models (RRM) can be a very efficient alternative for repeated measures analyses in *Jatropha curcas* breeding because they consider the genetic and residual covariance among harvests and allows fitting the genetic and non-genetic trajectories (plot and permanent environment) over time [11,12]. Besides that, RRM allow assessing genotype persistence, i.e., the capacity to maintain the yield performance over the years, which can be affected by biotic and abiotic effects [13–15]. Thus, this work aimed to apply the random regression technique and study the possibilities it offers for the analysis of repeated measures in *Jatropha curcas* breeding.

## Material and methods

### Experimental data

Seven hundred and thirty individuals from 73 half-sib families of *Jatropha curcas* were evaluated for grain yield (GY) trait (kg plant^-1^), in six harvests (2010 to 2015) (Supplementary material–S3 Table). The experiment was carried out in a randomized complete block design with two replications, five plants per plot, and spacing of 4 m between rows and 2 m between trees. The experiment was conducted in the experimental field of Embrapa Cerrados, located in Planaltina, Distrito Federal—Brazil (15°35’30” S and 47°42’30” W; 1007 m asl). All management practices were based on [16].

### Statistical analyses

The time of the harvests must be scaled to range from -1 to +1 in order to use Legendre polynomials. The scaling formula is given below [12]:

$$t_{x}=-1+2[\left(h_{x}-h_{min}\right)/\left(h_{max}-h_{min}\right)],$$

where *h*_*x*_ refers to the time of the harvest *x*; *h*_*min*_ is the time of the first harvest (2010); and, *h*_*max*_ is time of the last harvest (2015).

Variance components were estimated by restricted maximum likelihood [17], and genetic values were predicted by best linear unbiased prediction [18], according to [19]. Random regression models were fitted through Legendre polynomials, considering all possible degrees of fit for each random effect, using the following general model:

$$Y_{ijkl}=R_{k}+b_{M}\phi_{ijM}+\sum_{m=0}^{M}g_{im}\phi_{ijm}+\sum_{m=0}^{M}p_{ikm}\phi_{ijm}+\sum_{m=0}^{M}s_{ik}\phi_{ijm}+\varepsilon_{ijkl},$$

where *Y*_*ijkl*_ is the *i*^*th*^ individual (*i = 1*, *2*, *…*, *730*) on the *j*^th^ harvest (*j = 1*, *2*, *…*, *6*) on the *k*^th^ replication (*k = 1*, *2*) on the *l*^*th*^ plot (*l = 1*, *2*, *…*, *146*); *R*_*k*_ is the fixed effect of replication;. *b*_*M*_ is the fixed regression coefficient fitted through the fifth degree of Legendre polynomials to the common average trajectory of progenies. The random effects, *g*_*im*_, *p*_*ikm*_, and *s*_*ik*_ are the random regression coefficients for the Legendre polynomials of degree *m* for the genetic, permanent environment, and plot effects, respectively. *ϕ*_*ijm*_ is the *m*^th^ Legendre polynomial for the *j*^th^ harvest from the *i*^th^ individual; *m* is the fit degree, ranging from *M* = 0 to *M* = 5, of the Legendre polynomial for the genetic, permanent environment, and plot effects, respectively; and *ε*_*ijkl*_ is the residual random effect associated with *Y*_*ijkl*_.

In the matrix notation, the above model is described as follows:

y=Xb+Zg+Wp+Qs+e,

where *y* is the phenotypic data vector; *b* is the vector of the effects of measurement-replication combinations (assumed to be fixed) added to the overall mean; *g* is the vector of the individual genetic effects (assumed as random); *p* is the vector of the permanent environmental effects (assumed as random); *s* is the vector of the plot effects (assumed as random); and *e* is the vector of residuals (random). *X*, *Z*, W and *Q* refer to the incidence matrices for these effects.

In this model, *g*~*N*(0, *K*_*g*_⊗*I*), *p*~*N*(0, *K*_*p*_⊗*I*), *s*~*N*(0, *K*_*s*_⊗*I*), and *e*~*N*(0, *R*); where *K*_*g*_, *K*_*p*_, and *K*_*s*_ are the covariance matrices for genetic, permanent environment, and plot effects, respectively; ⊗ denotes the Kronecker product; *I* is an identity matrix with appropriate order to the respective random effect; and *R* refers to the matrix of residual covariances. Different residual covariance structures (homogeneous, diagonal, and unstructured) were tested.

The polynomial order in random regression models was selected using the Bayesian information criterion (BIC) [20], as follows:

BIC=−2LogL+pLog[n−r(x)]

where *LogL* is the logarithm of the maximum *(L)* of the residual likelihood function, *p* is the number of estimated parameters, *n* is the number of observations, and *r*(*x*) is the rank of the incidence matrix of fixed effect.

The significance of the genetic, permanent environment and plot effects was tested using the likelihood ratio test (LRT) [21], as follows:

$$LRT={-2(LogL-LogL}_{R}),$$

where *LogL*_*R*_ is the logarithm of the maximum (*L*_*R*_) of the residual likelihood function of the reduced model (without genetic or permanent environmental or plot effects).

Variance components estimates (${\hat{\sigma }}_{x}^{2}$) and the predicted genetic values (${\tilde{g}}_{ij}$), on the original scale, were obtained by the following expressions [22]:

$$\sigma_{x}^{2}=\phi_{ijm}K_{x}{\phi_{ijm}}^{\prime },\;\mathrm{and}$$

$${\tilde{g}}_{ij}=\sum_{m=0}^{M}\alpha_{im}\phi_{ijm},$$

where *x* refers to the genetic or permanent environmental or plot covariance matrices.

Phenotypic variance (${\hat{\sigma }}_{phen}^{2}$), individual heritability between progenies ($h_{g}^{2}$) and selective accuracy ($r_{\hat{g}g}$) were obtained by the following expressions [23]:

$${\hat{\sigma }}_{phen}^{2}={\hat{\sigma }}_{g}^{2}+{\hat{\sigma }}_{p}^{2}+{\hat{\sigma }}_{s}^{2}+{\hat{\sigma }}_{res}^{2},$$

$$h_{g}^{2}={\hat{\sigma }}_{g}^{2}/{\hat{\sigma }}_{phen}^{2},\;\mathrm{and}$$

$$r_{\hat{g}g}=\sqrt{1-(\phi_{ijm}PEV{\phi_{ijm}}^{\prime }/{\hat{\sigma }}_{g}^{2})},$$

where ${\hat{\sigma }}_{res}^{2}$ is the residual variance and *PEV* is the prediction error variance extracted from the diagonal of generalized inverse of the coefficient matrix of the mixed model equations.

The eigenfunction (*Ψ*_*f*_) of the genetic coefficient covariance matrix, aiming to evaluated the genotype x measurement interaction, was calculated by the following expression [22]:

$$\Psi_{f}=\sum_{m=0}^{M}{(c}_{\Psi_{f}}{)}_{m}\Phi_{m},$$

where ${(c}_{\Psi_{f}}{)}_{m}$ is the *m*^*th*^ element of the *f*^*th*^ eigenvector of *K*_*g*_, and *Φ*_*m*_ is the normalized value of the *m*^*th*^ Legendre polynomial.

The areas under the genetic trajectories (A), aiming to rank the clones, were obtained by the following expression [24,25]:

$$A=\mu +\int_{-1}^{1}\sum_{m=0}^{M}\alpha_{im}\phi_{ijm}x^{m}\;dx,$$

where *μ* is the phenotypic mean and where *x*^*m*^ is the is the harvest scaled. Genetic correlations (*ρ*_*g*_) between each pair of harvests were obtained by the following expression:

$$\rho_{g}=\frac{{\hat{\sigma }}_{g(ij)}}{\sqrt{{\hat{\sigma }}_{g(i)}^{2}{\hat{\sigma }}_{g(j)}^{2}}},$$

where ${\hat{\sigma }}_{g(ij)}$, is the genetic covariance between progenies for the pair of harvests *i* and *j*; ${\hat{\sigma }}_{g(i)}^{2}$ and ${\hat{\sigma }}_{g(j)}^{2}$ are the genetic variance between progenies for the harvests *i* and *j*, respectively.

Statistical analyses were performed using the ASReml 4.1 [19] and R [26] software.

## Results

According to the BIC, the best RRM was the one of order two for the genetic effects, order five for the plot effects, and order one for the permanent environment effects; with diagonal residual variance structure (Table 1 and Supplementary material—S1 Table). Thus, this model was adopted to estimate the variance components and predict the genetic values. When the models without genetic, plot or permanent environmental effects were tested by the LRT, the significance of genetic, plot, and permanent environmental effects (p-value < 0.01) was detected (LRT equal to -43.19, -1450.28, and -361.98, respectively).

**Table 1:** ASReml output for some models that converged for the grain yield trait evaluated in 73 half-sib *Jatropha curcas* progenies.

Polynomial degree			df	It	LogL	Parameters				BIC
Gen.	Plot	Perm.				Gen.	Plot.	Perm.	Res.	
0	5	0	4298	39	2570.4	1	21	1	6	-4980.9
1	5	0	4298	35	2578.52	3	21	1	6	-4989.9
2	5	0	4298	37	2585.77	6	21	1	6	-4993.5
3	5	0	4298	43	2590.15	10	21	1	6	-4987.7
4	5	0	4298	46	2596.27	15	21	1	6	-4981.8
0	5	1	4298	14	2683.74	1	21	3	6	-5207.6
1	5	1	4298	23	2691.81	3	21	3	6	-5216.5
**2**	**5**	**1**	**4298**	**41**	**2699.04**	**6**	**21**	**3**	**6**	**-5220.1**
3	5	1	4298	41	2703.27	10	21	3	6	-5214
4	5	1	4298	46	2709.36	15	21	3	6	-5208
0	0	0	4298	8	1021.81	1	1	1	6	-1956.4

The selected model by Bayesian information criterion (BIC) was indicated in bold. Gen: Genetic effect; Plot: Plot effect; Perm: Permanent environmental effect; Res: Residual effect; df: Degrees of freedom; Ite: Number of iterations; and, LogL: Logarithm of the restricted maximum likelihood function. The complete list of models that converged are presented in the S1 Table–Supplementary material.

The genetic variance was not constant through the harvests (Table 2). The estimates increased from the first harvest (0.0034) to the last harvest (0.3232). Similar patterns were found for permanent environmental variance (0.0035 to 0.1064) and plot variance (0.0042 to 0.2165). In addition, the residual variance rate in the first harvest was 0.0020. After a steady increase, it reached 0.2154 in the last harvest. Consequently, the phenotypic variance presented a steady increase from the first harvest (0.0133) to the sixth harvest (0.8616). In addition, the individual heritability estimates ranged from 0.05 (2012) to 0.37 (2015) (Table 1). The selective accuracy presented high magnitudes in all harvests, and demonstrated an upward trend over time, ranging from 0.79 in the first harvest to 0.86 in the last harvest (Table 2).

**Table 2:** Estimates of variance components and genetic parameters for the grain yield trait evaluated in 73 half-sib *Jatropha curcas* progenies.

Harvest	${\hat{\sigma }}_{g}^{2}$	${\hat{\sigma }}_{s}^{2}$	${\hat{\sigma }}_{p}^{2}$	${\hat{\sigma }}_{res}^{2}$	${\hat{\sigma }}_{phen}^{2}$	$h_{g}^{2}$	$\bar{r_{\hat{g}g}}$	μ
**2010**	0.0034 (0.0045)	0.0042 (0.1061)	0.0035 (0.0002)	0.002 (0.0006)	0.0133	0.25	0.79	0.201
**2011**	0.0042 (0.0029)	0.0338 (0.0094)	0.0083 (0.0006)	0.0231 (0.0015)	0.0696	0.06	0.81	0.528
**2012**	0.0172 (0.0059)	0.1879 (0.0043)	0.0209 (0.0018)	0.1079 (0.0068)	0.3341	0.05	0.83	1.488
**2013**	0.0565 (0.0151)	0.2736 (0.0213)	0.0415 (0.0038)	0.0988 (0.0068)	0.4705	0.12	0.85	1.314
**2014**	0.1466 (0.0390)	0.2406 (0.0124)	0.0700 (0.0067)	0.1368 (0.0100)	0.5942	0.24	0.85	2.050
**2015**	0.3232 (0.0927)	0.2165 (0.7173)	0.1064 (0.0104)	0.2154 (0.0159)	0.8616	0.37	0.86	2.656

${\hat{\sigma }}_{g}^{2}$: Genetic variance between families; ${\hat{\sigma }}_{s}^{2}$: Plot variance; ${\hat{\sigma }}_{p}^{2}$: Permanent environmental variance; ${\hat{\sigma }}_{res}^{2}$: Residual variance; ${\hat{\sigma }}_{phen}^{2}$: Phenotypic variance; $h_{g}^{2}$: Individual heritability; $\bar{r_{\hat{g}g}}$: Mean selective accuracy; and μ: Phenotypic mean.

Values between parentheses represents the standard errors for the variance components.

**Fig 1:**
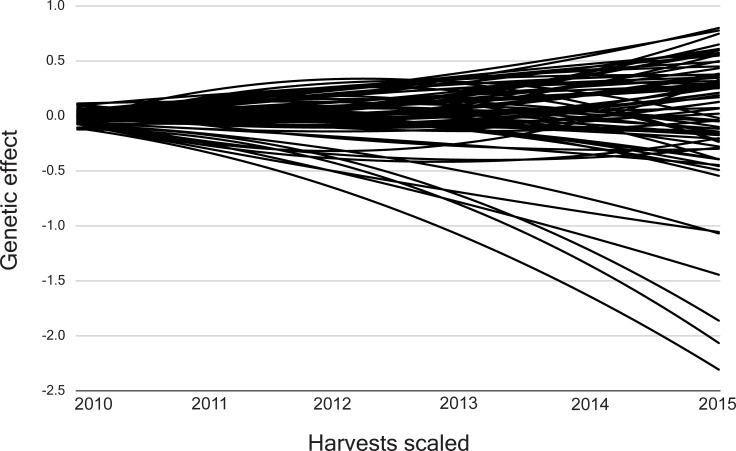
Trajectories for the 73 half-sib *Jatropha curcas* progenies evaluated for the grain yield trait.

The genetic trajectories exhibited a non-linear form and genotypic plasticity for the 73 half-sib *Jatropha curcas* progenies (Fig 1). They presented a continuous deviation in the first three harvests and then, different forms of deviation until the sixth harvest.

**Fig 2:**
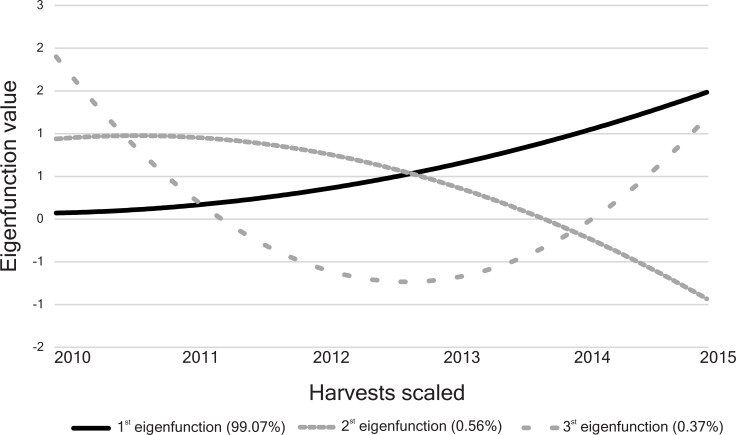
Eigenfunctions for the 73 half-sib *Jatropha curcas* progenies evaluated for the grain yield trait. Their proportional eigenvalues for the genetic covariance function are in parentheses.

The first eigenfunction presented a concave crescent trajectory and accounted for more than 99% of the genetic variance (eigenvalue = 0.1460) (Fig 2). The second and third eigenfunctions explain only 0.6% (eigenvalue = 0.0008) and 0.4% (eigenvalue = 0.0005) of the genetic variability, respectively. The trajectory of the second eigenfunction was continuously over the third harvest and then decreased, whereas the third eigenfunction showed a concave deviation, with decreasing values until the third harvest and rising values until the last harvest (Fig 2).

**Fig 3:**
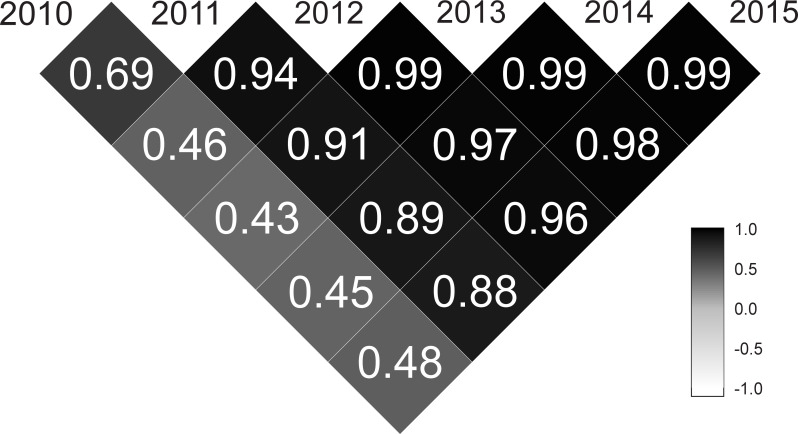
Genetic correlations between pairs of harvests (2010–2015) for the 73 half-sib *Jatropha curcas* progenies evaluated for the grain yield trait.

The area under the genetic trajectories was calculated for genotype ranking, and the highest values represent those progenies with the best overall performance over time. In this case, different values of areas were found for the 73 half-sib *Jatropha curcas* progenies. They ranged from -0.5879 to 2.0520, and the top ten families (those with larger area under the genetic trajectories, from the first to the tenth families selected) were: 6, 70, 48, 16, 10, 1, 34, 39, 15, 29, and 54. The complete rank are presented in the supplementary material (S2 Table). In addition, the genetic correlations between pairs of harvests presented moderate magnitudes (0.33 < *ρ*_*g*_ < 0.66) in the 2010–2012, 2010–2013, 2010–2014, 2010–2015; high magnitudes (0.67 < *ρ*_*g*_ < 0.89) in the 2010–2011, 2011–2014, and 2011–2015; and very high magnitudes (*ρ*_*g*_ > 0.90) in the remaining pairs of harvests (Fig 3).

## Discussion

Among the various criteria for selection of models, the BIC is prominent, because it is a consistent criterion. The selected model fit diagonal residual variance structure (*i*.*e*., one residual variance for each harvest). Considering the other random effects of the model (genetic, permanent, and plot), a total of 36 covariance components were estimated. The RRM are equivalent to the covariance functions and can be considered a reduced and simplified multiple-trait model, which allows the same parameters of interest (heritability and genetic correlation among all pairs of harvests) to be obtained, but with lower parameterization and with less computational effort [23,27].

Since there are reliable estimates of variance components, they allow the prediction of genetic values of individuals evaluated at different ages (and with different numbers of ages evaluated) and the projection of these genetic values for a common age, for ordering and selection purposes. Besides that, Legendre polynomials have been used to model growth curves in perennial plant breeding [14,28].

According to the LRT, there was genetic variability among the *Jatropha curcas* families. Besides that, the plot and permanent environmental effects are statistically significant (p-value < 0.01), i.e., they differ from zero. The significance of genetic effects shows the potential of this population and allow the selection of superior families, even with a restricted genetic basis explored in *Jatropha curcas* breeding in Brazil [6,29]. It is worth mentioning that the RRM allows fitting the permanent environmental effect. On the other hand, the multiple-trait model does not allow fitting this effect, since it assumes that the same trait in different harvest is a different trait [10].

The RRM is mainly focused on visualizing trajectories and covariance functions over time. Therefore, the variance components and genetic parameters can vary over the harvests. In this study, it is generally observed an increasing trend in the variance components and genetic parameters over time. Such pattern was also observed by [1,10,30].

The individual heritability estimates decreased from the first to the third harvest. Then, there was an upward trend until the sixth harvest, which indicates that the third harvest is more affected by the environmental conditions. According to [31], the heritabilities are classified as low ($h_{g}^{2}<0.30)$, except for the last one, which was classified as moderate ($0.30<h_{g}^{2}<0.50$). Similar results were reported in other studies with *Jatropha curcas* [10,30]. Besides that, the variation in the individual heritability estimates was like the variation in the variance components estimates.

Upward trend in genetic variances and heritabilities along the time, are biologically consistent. Such trends can be related to the fact that *Jatropha curcas* genotypes are more sensitive to environmental variations in the early stages of growth [1,30], due to the higher genotype × measurement interaction. Indeed, in this work, the genotype × measurement interaction effect was significant. Further, it is related with the fact that the metabolism of young perennials often privilege vegetative rather than reproductive growth [32], which leads to an uneven production in the early harvests. Then, given the temporal trend of the genetic parameters and genetic values, the selection for the GY trait should consider several harvests (three to six, according to [11] for an accurate genetic selection).

According to [33], the selective accuracies were classified as high in all harvests (0.70 < $r_{\hat{g}g}$ < 0.90). In fact, when compared with other models, including the multiple-trait models, the selective accuracy by RRM has presented values considered higher [34]. This is probably because RRM does not require pre-adjustment of weights at standard ages, which may provide gains in selective accuracy [35].

The genetic trajectories describe the genetic values of each family over time and encompass the six harvests evaluated in this study. The RRM can predict the genetic value for any family in any time (between the first and sixth harvest). The trajectories demonstrated that the families presented similar performance in the first harvests, which reveals that a precocious selection tends to be less efficient. The genetic correlations reinforce the inefficiency in earlier selection. The genetic correlations between the pairs of harvest presented very high values (> 0.90) only between the last harvests. In addition [9], showed that ten harvests are necessary for an accurate genetic selection for the GY trait in *Jatropha curcas*.

The RRM fitted through Legendre polynomials allows obtain the eigenfunctions and eigenvalues [22]. According to those authors, the eigenfunctions are similar to the eigenvectors of the principal component analyses and can be interpreted as proportional to the amount of genetic variation in the population corresponding to that eigenfunction. The first eigenfunction clustered general adaptability genes equally expressed in all harvests [14]. This can be interpreted as the genetic correlation among the harvests. The second and third eigenfunctions showed small eigenvalues and represent deformations for which there is little (or no) genetic variation [22]. Perennial plants usually present great variations in productivity in the initial harvests, since many genes expressed in that period are associated with the formation of vegetative organs [32]. This fact is typical of perennials and is indicated to occur in *Jatropha curcas* [30].

The genetic trajectories of the 73 *Jatropha curcas* progenies reinforced the presence of genotype × measurement interaction, once their trajectories are non-linear and intersect each other, which implies a different ranking in each harvest. In addition, the trajectories could also be interpreted as genetic variability. The more distant the genetic trajectories from each other, the more genetically distinct are the progenies [23].

In this study, genotype ranking was performed based on the areas under the genetic trajectories. Therefore, genetic selection was based on the higher area under the genetic trajectories. The advantage of this strategy is that selection response can be predicted not only in the genotypic expression in any harvest but also in quantifying the environmental sensitivity of the trait through the genetic trajectories (robustness or responsiveness to changes in the environment). Besides that, it can be used for any number of harvests [36].

The RRM can be used to help describing the observed phenotypic over time efficiently and allows genetic selection based on adaptability, stability and yield performance [14,36–38]. The main advantage of the RRM is the fact that it is biologically realistic through their emphasis on dynamic aspects of the phenotype and for allowing breeding questions on plasticity, adaptability, stability and yield performance to be answered [36]. Thus, our results suggest that RRM fitted through Legendre polynomials can be efficiently used in *Jatropha curcas* breeding programs.

## Supporting information

S1 Table ASReml output for the models that converged for the grain yield trait evaluated in 73 half-sib *Jatropha curcas* progenies. P.D. Gen: polynomial degree for the genetic effect; P.D. Plot: polynomial degree for the plot effect; P.D. Perm: polynomial degree for the permanent environment effect; DF: degrees of freedom; LogL: logarithm of the maximum of the restricted likelihood function; Gen. P.: total number of genetic covariances estimated; Plot P.: total number of plot covariances estimated; Perm. P.: total number of permanent environment covariance estimated; Res. P: total number of residual covariances estimated; and BIC: Bayesian information criterion. The selected model by BIC was indicated in bold.
(DOCX)

S2 Table Ranking of the 73 half-sib *Jatropha curcas* progenies for the grain yield trait based on the areas under the trajectories (A). (DOCX)

S3 Table Data of the 73 half-sib *Jatropha curcas* progenies for the grain yield trait. (DOCX)
