# Supplementary material for: Random regression for modeling yield genetic trajectories in Jatropha curcas breeding
Source: PLoS One. 2020 Dec 23;15(12):e0244021. doi: 10.1371/journal.pone.0244021 (PMC7757908; doi:10.1371/journal.pone.0244021)
Supplement: S1 Table — P.D. Gen: polynomial degree for the genetic effect; P.D. Plot: polynomial degree for the plot effect; P.D. Perm: polynomial degree for the permanent environment effect; DF: degrees of freedom; LogL: logarithm of the maximum of the restricted likelihood function; Gen. P.: total number of genetic covariances estimated; Plot P.: total number of plot covariances estimated; Perm. P.: total number of permanent environment covariance estimated; Res. P: total number of residual covariances estimated; and BIC: Bayesian information criterion. The selected model by BIC was indicated in bold. (DOCX) [file pone.0244021.s001.docx]

**Random regressions for modeling yield genetic trajectories in** ***Jatropha curcas* breeding**

Marco Antônio Peixoto^1^, Rodrigo Silva Alves^2^, Igor Ferreira Coelho^1^, Jeniffer Santana Pinto Coelho Evangelista^1^, Marcos Deon Vilela de Resende^3^, João Romero do Amaral Santos de Carvalho Rocha^1^, Fabyano Fonseca e Silva^1^, Bruno Gâlveas Laviola^4^, Leonardo Lopes Bhering^1^

^1^ Universidade Federal de Viçosa, Viçosa, MG, Brazil.

^2^ INCT Café/Universidade Federal de Viçosa, Viçosa, MG, Brazil.

^3^ Embrapa Café/Universidade Federal de Viçosa, Viçosa, MG, Brazil.

^4^ Embrapa Agroenergia, Brasília, DF, Brazil.

**Corresponding author:** Leonardo Lopes Bhering, leonardo.bhering@ufv.br

**Table S1.** ASReml output for all models that converged for the grain yield trait evaluated in 73 half-sib *Jatropha curcas* progenies. P.D. Gen: polynomial degree for the genetic effect; P.D. Plot: polynomial degree for the plot effect; P.D. Perm: polynomial degree for the permanent environment effect; DF: degrees of freedom; LogL: logarithm of the restricted maximum likelihood function; Gen. P.: total number of genetic covariances estimated; Plot P.: total number of plot covariances estimated; Perm. P.: total number of permanent environment covariance estimated; Res. P: total number of residual covariances estimated; and BIC: Bayesian information criterion. The selected model by BIC was indicated in bold.

| **Fixed effect order** | **P.D. Gen** | **P.D. Plot** | **P.D. Perm** | **DF** | **Iterations** | **LogL** | **Geno. P** | **Plot. P** | **Perm. P** | **Res. P** | **BIC** |
| --- | --- | --- | --- | --- | --- | --- | --- | --- | --- | --- | --- |
| 5 | 0 | 0 | 0 | 4298 | 9 | 419.80 | 1 | 1 | 1 | 1 | -752.39 |
| 5 | 0 | 1 | 0 | 4298 | 69 | 842.16 | 1 | 3 | 1 | 1 | -1589.86 |
| 5 | 1 | 0 | 0 | 4298 | 9 | 1889.54 | 3 | 1 | 1 | 1 | -3684.62 |
| 5 | 2 | 0 | 0 | 4298 | 9 | 1952.19 | 6 | 1 | 1 | 1 | -3799.02 |
| 5 | 3 | 0 | 0 | 4298 | 9 | 2002.74 | 10 | 1 | 1 | 1 | -3885.58 |
| 5 | 4 | 0 | 0 | 4298 | 10 | 2069.50 | 15 | 1 | 1 | 1 | -4000.94 |
| 5 | 5 | 0 | 0 | 4298 | 9 | 2176.16 | 21 | 1 | 1 | 1 | -4192.46 |
| 5 | 0 | 0 | 1 | 4298 | 13 | 1778.33 | 1 | 1 | 3 | 1 | -3469.46 |
| 5 | 1 | 0 | 1 | 4298 | 11 | 1941.65 | 3 | 1 | 3 | 1 | -3788.84 |
| 5 | 2 | 0 | 1 | 4298 | 10 | 2018.49 | 6 | 1 | 3 | 1 | -3931.62 |
| 5 | 3 | 0 | 1 | 4298 | 9 | 2084.01 | 10 | 1 | 3 | 1 | -4048.12 |
| 5 | 4 | 0 | 1 | 4298 | 10 | 2177.34 | 15 | 1 | 3 | 6 | -4216.62 |
| 5 | 5 | 0 | 1 | 4298 | 9 | 2323.17 | 21 | 1 | 3 | 6 | -4486.48 |
| 5 | 0 | 1 | 0 | 4298 | 11 | 2008.22 | 1 | 3 | 1 | 6 | -3921.98 |
| 5 | 1 | 1 | 0 | 4298 | 9 | 2016.82 | 3 | 3 | 1 | 6 | -3931.91 |
| 5 | 2 | 1 | 0 | 4298 | 30 | 2089.43 | 6 | 3 | 1 | 6 | -4066.23 |
| 5 | 3 | 1 | 0 | 4298 | 29 | 2147.36 | 10 | 3 | 1 | 6 | -4167.56 |
| 5 | 4 | 1 | 0 | 4298 | 30 | 2227.97 | 15 | 3 | 1 | 6 | -4310.61 |
| 5 | 5 | 1 | 0 | 4298 | 30 | 2364.95 | 21 | 3 | 1 | 6 | -4562.77 |
| 5 | 2 | 1 | 1 | 4298 | 31 | 2097.54 | 6 | 3 | 3 | 6 | -4082.45 |
| 5 | 3 | 1 | 1 | 4298 | 30 | 2160.43 | 10 | 3 | 3 | 6 | -4193.70 |
| 5 | 4 | 1 | 1 | 4298 | 30 | 2252.03 | 15 | 3 | 3 | 6 | -4358.73 |
| 5 | 5 | 1 | 1 | 4298 | 30 | 2404.12 | 21 | 3 | 3 | 6 | -4641.11 |
| 5 | 0 | 2 | 0 | 4298 | 11 | 2117.10 | 1 | 6 | 1 | 6 | -4128.84 |
| 5 | 1 | 2 | 0 | 4298 | 34 | 2124.41 | 3 | 6 | 1 | 6 | -4136.19 |
| 5 | 2 | 2 | 0 | 4298 | 30 | 2130.94 | 6 | 6 | 1 | 6 | -4138.35 |
| 5 | 3 | 2 | 0 | 4298 | 30 | 2203.62 | 10 | 6 | 1 | 6 | -4269.18 |
| 5 | 4 | 2 | 0 | 4298 | 33 | 2288.23 | 15 | 6 | 1 | 6 | -4420.23 |
| 5 | 5 | 2 | 0 | 4298 | 33 | 2426.72 | 21 | 6 | 1 | 6 | -4675.41 |
| 5 | 0 | 2 | 1 | 4298 | 20 | 2130.32 | 1 | 6 | 3 | 6 | -4155.28 |
| 5 | 1 | 2 | 1 | 4298 | 33 | 2137.66 | 3 | 6 | 3 | 6 | -4162.69 |
| 5 | 2 | 2 | 1 | 4298 | 29 | 2144.00 | 6 | 6 | 3 | 6 | -4164.47 |
| 5 | 3 | 2 | 1 | 4298 | 36 | 2226.88 | 10 | 6 | 3 | 6 | -4315.70 |
| 5 | 4 | 2 | 1 | 4298 | 34 | 2325.51 | 15 | 6 | 3 | 6 | -4494.79 |
| 5 | 5 | 2 | 1 | 4298 | 35 | 2480.93 | 21 | 6 | 3 | 6 | -4783.83 |
| 5 | 0 | 3 | 0 | 4298 | 13 | 2201.73 | 1 | 10 | 1 | 6 | -4283.56 |
| 5 | 1 | 3 | 0 | 4298 | 37 | 2209.76 | 3 | 10 | 1 | 6 | -4292.36 |
| 5 | 2 | 3 | 0 | 4298 | 42 | 2216.03 | 6 | 10 | 1 | 6 | -4294.00 |
| 5 | 3 | 3 | 0 | 4298 | 32 | 2221.65 | 10 | 10 | 1 | 6 | -4290.70 |
| 5 | 4 | 3 | 0 | 4298 | 43 | 2307.99 | 15 | 10 | 1 | 6 | -4445.22 |
| 5 | 5 | 3 | 0 | 4298 | 56 | 2453.21 | 21 | 10 | 1 | 6 | -4713.86 |
| 5 | 0 | 3 | 1 | 4298 | 11 | 2227.64 | 1 | 10 | 3 | 6 | -4335.38 |
| 5 | 1 | 3 | 1 | 4298 | 36 | 2235.49 | 3 | 10 | 3 | 6 | -4343.82 |
| 5 | 2 | 3 | 1 | 4298 | 49 | 2241.53 | 6 | 10 | 3 | 6 | -4345.00 |
| 5 | 3 | 3 | 1 | 4298 | 34 | 2247.28 | 10 | 10 | 3 | 6 | -4341.96 |
| 5 | 4 | 3 | 1 | 4298 | 41 | 2349.70 | 15 | 10 | 3 | 6 | -4528.64 |
| 5 | 5 | 3 | 1 | 4298 | 41 | 2513.32 | 21 | 10 | 3 | 6 | -4834.08 |
| 5 | 0 | 4 | 0 | 4298 | 20 | 2309.02 | 1 | 15 | 1 | 6 | -4479.98 |
| 5 | 1 | 4 | 0 | 4298 | 35 | 2317.36 | 3 | 15 | 1 | 6 | -4489.39 |
| 5 | 2 | 4 | 0 | 4298 | 39 | 2324.38 | 6 | 15 | 1 | 6 | -4492.53 |
| 5 | 3 | 4 | 0 | 4298 | 44 | 2329.53 | 10 | 15 | 1 | 6 | -4488.30 |
| 5 | 4 | 4 | 0 | 4298 | 55 | 2334.75 | 15 | 15 | 1 | 6 | -4480.57 |
| 5 | 5 | 4 | 0 | 4298 | 46 | 2489.33 | 21 | 15 | 1 | 6 | -4767.93 |
| 5 | 0 | 4 | 1 | 4298 | 12 | 2363.29 | 1 | 15 | 3 | 6 | -4588.52 |
| 5 | 1 | 4 | 1 | 4298 | 31 | 2371.82 | 3 | 15 | 3 | 6 | -4598.31 |
| 5 | 2 | 4 | 1 | 4298 | 52 | 2378.75 | 6 | 15 | 3 | 6 | -4601.27 |
| 5 | 3 | 4 | 1 | 4298 | 43 | 2384.19 | 10 | 15 | 3 | 6 | -4597.62 |
| 5 | 4 | 4 | 1 | 4298 | 41 | 2389.38 | 15 | 15 | 3 | 6 | -4589.83 |
| 5 | 5 | 4 | 1 | 4298 | 42 | 2567.22 | 21 | 15 | 3 | 6 | -4923.71 |
| 5 | 0 | 5 | 0 | 4298 | 39 | 2570.40 | 1 | 21 | 1 | 6 | -4980.94 |
| 5 | 1 | 5 | 0 | 4298 | 35 | 2578.52 | 3 | 21 | 1 | 6 | -4989.91 |
| 5 | 2 | 5 | 0 | 4298 | 37 | 2585.77 | 6 | 21 | 1 | 6 | -4993.51 |
| 5 | 3 | 5 | 0 | 4298 | 43 | 2590.15 | 10 | 21 | 1 | 6 | -4987.74 |
| 5 | 4 | 5 | 0 | 4298 | 46 | 2596.27 | 15 | 21 | 1 | 6 | -4981.81 |
| 5 | 0 | 5 | 1 | 4298 | 14 | 2683.74 | 1 | 21 | 3 | 6 | -5207.62 |
| 5 | 1 | 5 | 1 | 4298 | 23 | 2691.81 | 3 | 21 | 3 | 6 | -5216.49 |
| **5** | **2** | **5** | **1** | **4298** | **41** | **2699.04** | 6 | 21 | 3 | **6** | **-5220.05** |
| 5 | 3 | 5 | 1 | 4298 | 41 | 2703.27 | 10 | 21 | 3 | 6 | -5213.98 |
| 5 | 4 | 5 | 1 | 4298 | 46 | 2709.36 | 15 | 21 | 3 | 6 | -5207.99 |
| 5 | 0 | 0 | 0 | 4298 | 8 | 1021.81 | 1 | 1 | 1 | 6 | -1956.42 |
