# Supplementary material for: Random regression for modeling yield genetic trajectories in Jatropha curcas breeding
Source: PLoS One. 2020 Dec 23;15(12):e0244021. doi: 10.1371/journal.pone.0244021 (PMC7757908; doi:10.1371/journal.pone.0244021)
Supplement: S2 Table — (DOCX) [file pone.0244021.s002.docx]

**Table S2.** Ranking of the 73 half-sib *Jatropha curcas* progenies for the grain yield trait based on the areas under the trajectories (A).

| **Rank** | **Progeny** | **A** | **Rank** | **Progeny** | **A** |
| --- | --- | --- | --- | --- | --- |
| 1 | 6 | 0.6869 | 37 | 14 | 0.0798 |
| 2 | 70 | 0.6254 | 38 | 3 | 0.0762 |
| 3 | 48 | 0.5805 | 39 | 17 | 0.0628 |
| 4 | 16 | 0.5791 | 40 | 64 | 0.0491 |
| 5 | 10 | 0.5636 | 41 | 38 | 0.0468 |
| 6 | 1 | 0.5436 | 42 | 18 | 0.0444 |
| 7 | 34 | 0.5326 | 43 | 58 | 0.0036 |
| 8 | 39 | 0.5245 | 44 | 13 | -0.0159 |
| 9 | 15 | 0.4982 | 45 | 46 | -0.0165 |
| 10 | 29 | 0.4458 | 46 | 69 | -0.0295 |
| 11 | 54 | 0.4291 | 47 | 43 | -0.0687 |
| 12 | 41 | 0.4110 | 48 | 22 | -0.0699 |
| 13 | 73 | 0.3981 | 49 | 25 | -0.0791 |
| 14 | 35 | 0.3968 | 50 | 52 | -0.0823 |
| 15 | 9 | 0.3960 | 51 | 60 | -0.0919 |
| 16 | 4 | 0.3936 | 52 | 7 | -0.1215 |
| 17 | 56 | 0.3874 | 53 | 30 | -0.1481 |
| 18 | 37 | 0.3796 | 54 | 44 | -0.1529 |
| 19 | 2 | 0.3548 | 55 | 21 | -0.1574 |
| 20 | 61 | 0.3222 | 56 | 63 | -0.1737 |
| 21 | 67 | 0.3084 | 57 | 20 | -0.1962 |
| 22 | 62 | 0.3039 | 58 | 12 | -0.2225 |
| 23 | 11 | 0.2994 | 59 | 50 | -0.2379 |
| 24 | 32 | 0.2533 | 60 | 49 | -0.2434 |
| 25 | 42 | 0.2502 | 61 | 28 | -0.2440 |
| 26 | 5 | 0.2464 | 62 | 51 | -0.2626 |
| 27 | 65 | 0.2435 | 63 | 59 | -0.3014 |
| 28 | 53 | 0.2373 | 64 | 55 | -0.3991 |
| 29 | 47 | 0.2357 | 65 | 8 | -0.4681 |
| 30 | 68 | 0.2041 | 66 | 66 | -0.6184 |
| 31 | 36 | 0.1960 | 67 | 23 | -0.6358 |
| 32 | 24 | 0.1755 | 68 | 33 | -0.9092 |
| 33 | 40 | 0.1718 | 69 | 57 | -1.1782 |
| 34 | 45 | 0.1568 | 70 | 19 | -1.3463 |
| 35 | 71 | 0.1282 | 71 | 26 | -1.3712 |
| 36 | 72 | 0.1051 | 72 | 31 | -1.5316 |
|  |  |  | 73 | 27 | -1.9530 |
