# Supplementary material for: Random regression for modeling yield genetic trajectories in Jatropha curcas breeding
Source: PLoS One. 2020 Dec 23;15(12):e0244021. doi: 10.1371/journal.pone.0244021 (PMC7757908; doi:10.1371/journal.pone.0244021)
Supplement: S3 Table — (DOCX) [file pone.0244021.s003.docx]

**Table S3.** Data from the 73 half-sib *Jatropha curcas* progenies for the grain yield trait.

| ID | Progeny | Block | Plot | 2010 | 2011 | 2012 | 2013 | 2014 | 2015 |
| --- | --- | --- | --- | --- | --- | --- | --- | --- | --- |
| 1 | 1 | 1 | 11 | 0.18045 | 0.61617 | 0.8916 | 1.7237 | 1.9728 | 1.885 |
| 2 | 1 | 1 | 11 | 0.16645 | 0.78746 | 1.7988 | 1.3098 | 2.5904 | 1.82 |
| 3 | 1 | 1 | 11 | 0.0814 | 0.99686 | 2.0024 | 1.4558 | 2.21 | 2.79 |
| 4 | 1 | 1 | 11 | 0.2033 | 1.11 | 1.7382 | 2.2038 | 2.4825 | 3.315 |
| 5 | 1 | 1 | 11 | 0.153 | 0.91575 | 1.7962 | 1.9983 | 2.4745 | 4.39 |
| 6 | 1 | 2 | 12 | 0.1259 | 0.39758 | 1.2673 | 2.3248 | 2.9649 | 3.4 |
| 7 | 1 | 2 | 12 | 0.0278 | 0.40661 | 1.1562 | 2.7454 | 3.1882 | 3.668 |
| 8 | 1 | 2 | 12 | 0.2866 | 0.71016 | 1.4785 | 2.6926 | 2.2984 | 3.002 |
| 9 | 1 | 2 | 12 | 0.2394 | 0.51557 | 1.0246 | 2.4674 | 2.7032 | 3.222 |
| 10 | 1 | 2 | 12 | NA | 0.31931 | 0.5701 | 2.3857 | 2.1196 | 2.838 |
| 11 | 2 | 1 | 21 | 0.107 | 1.23939 | 1.4615 | 0.58334 | 3.0732 | 2.415 |
| 12 | 2 | 1 | 21 | 0.20162 | 1.05472 | 1.6153 | 0.6528 | 2.5216 | 2.35 |
| 13 | 2 | 1 | 21 | 0.13966 | 1.63931 | 2.2228 | 1.06434 | 2.9415 | 3.16 |
| 14 | 2 | 1 | 21 | 0.1968 | 1.5899 | 1.5323 | 1.2923 | 3.1824 | 2.634 |
| 15 | 2 | 1 | 21 | 0.17078 | 1.18223 | 1.5894 | 0.75279 | 2.561 | 2.194 |
| 16 | 2 | 2 | 22 | 0.0746 | 0.78678 | 1.5415 | 0.4238 | 1.6133 | 2.504 |
| 17 | 2 | 2 | 22 | 0.1612 | 0.77878 | 1.6074 | 0.8361 | 2.0636 | 2.786 |
| 18 | 2 | 2 | 22 | 0.1785 | 0.97701 | 2.0248 | 0.2847 | 2.2094 | 2.364 |
| 19 | 2 | 2 | 22 | 0.2742 | 0.92362 | 1.5526 | 0.6042 | 2.3723 | 2.918 |
| 20 | 2 | 2 | 22 | 0.1077 | 0.84182 | 1.0735 | 0.3544 | 1.9515 | 1.996 |
| 21 | 3 | 1 | 31 | 0.24736 | 0.89708 | 1.436 | 0.8644 | 0.8435 | 0.818 |
| 22 | 3 | 1 | 31 | 0.15584 | 0.67526 | 1.7906 | 0.8234 | 3.2995 | 2.008 |
| 23 | 3 | 1 | 31 | 0.20406 | 1.30514 | 1.9783 | 1.1728 | 3.3616 | 3.072 |
| 24 | 3 | 1 | 31 | 0.20808 | 1.44299 | 1.6417 | 0.987 | 2.6964 | 2.642 |
| 25 | 3 | 1 | 31 | 0.26532 | 0.85713 | 1.9479 | 0.9552 | 2.3434 | 2.378 |
| 26 | 3 | 1 | 31 | 0.10868 | 1.31846 | 1.6143 | 0.856 | 2.9211 | 2.718 |
| 27 | 3 | 2 | 32 | 0.0354 | 0.43397 | 0.848 | 0.78472 | 0.7491 | 1.104 |
| 28 | 3 | 2 | 32 | 0.1565 | 0.84653 | 1.7104 | 0.91746 | 1.8782 | 3.062 |
| 29 | 3 | 2 | 32 | 0.0958 | 0.60071 | 1.6134 | 0.79506 | 2.2138 | 2.478 |
| 30 | 3 | 2 | 32 | 0.2112 | 0.85441 | 2.1851 | 0.96519 | 2.5379 | 3.114 |
| 31 | 3 | 2 | 32 | 0.1653 | 0.34137 | 0.9492 | 0.81829 | 0.84 | 0.85 |
| 32 | 4 | 1 | 41 | 0.14234 | 1.03378 | 1.2384 | 1.0921 | 2.7764 | 2.68 |
| 33 | 4 | 1 | 41 | 0.1084 | 1.85778 | 1.1114 | 0.6835 | 3.54 | 2.056 |
| 34 | 4 | 1 | 41 | 0.14678 | 1.48349 | 1.927 | 0.5296 | 3.3199 | 2.162 |
| 35 | 4 | 1 | 41 | 0.12962 | 1.05489 | 1.3243 | 0.4131 | 1.8627 | 1.674 |
| 36 | 4 | 2 | 42 | 0.1384 | 0.73605 | 1.4225 | 1.149 | 2.3617 | 2.548 |
| 37 | 4 | 2 | 42 | 0.0977 | 0.66425 | 1.7854 | 1.7039 | 1.9589 | 3.64 |
| 38 | 4 | 2 | 42 | 0.0351 | 0.73261 | 2.0495 | 1.4464 | 2.2061 | 4.212 |
| 39 | 4 | 2 | 42 | 0.0652 | 0.62075 | 1.2495 | 0.9658 | 2.3279 | 3.386 |
| 40 | 4 | 2 | 42 | 0.0902 | 0.36634 | 1.3158 | 1.5424 | 1.8539 | 3.722 |
| 41 | 5 | 1 | 51 | 0.15595 | 1.10738 | 3.0284 | 1.45665 | 3.7122 | 2.128 |
| 42 | 5 | 1 | 51 | 0.07075 | 1.36506 | 1.9668 | 1.1948 | 2.6936 | 2.344 |
| 43 | 5 | 1 | 51 | 0.08728 | 0.6589 | 2.6994 | 1.12943 | 2.6919 | 2.278 |
| 44 | 5 | 1 | 51 | 0.10307 | 0.64694 | 2.3555 | 1.5382 | 1.3524 | 3.128 |
| 45 | 5 | 1 | 51 | 0.13079 | 0.57115 | 2.5848 | 1.14238 | 1.3635 | 2.38 |
| 46 | 5 | 2 | 52 | 0.0964 | 0.76628 | 2.1377 | 0.8514 | 1.8235 | 2.356 |
| 47 | 5 | 2 | 52 | 0.1876 | 0.43883 | 1.5443 | 0.863 | 1.754 | 3.152 |
| 48 | 5 | 2 | 52 | 0.0649 | 0.40767 | 2.929 | 0.8976 | 0.3477 | 0.969 |
| 49 | 5 | 2 | 52 | 0.0649 | 0.2108 | 2.2419 | 1.0185 | 2.2493 | 2.904 |
| 50 | 5 | 2 | 52 | 0.1214 | 0.44608 | 2.6222 | 1.3509 | 2.1611 | 3.328 |
| 51 | 6 | 1 | 61 | 0.30855 | 0.84461 | 2.5716 | 1.9756 | 4.2133 | 2.908 |
| 52 | 6 | 1 | 61 | 0.15626 | 0.42711 | 2.174 | 2.3571 | 3.2499 | 3.618 |
| 53 | 6 | 1 | 61 | 0.22528 | 1.01963 | 2.1553 | 2.9305 | 3.4934 | 3.294 |
| 54 | 6 | 1 | 61 | 0.38285 | 1.15192 | 2.3861 | 3.1884 | 2.9778 | 3.978 |
| 55 | 6 | 1 | 61 | 0.32811 | 0.76969 | 2.2403 | 2.7292 | 3.2629 | 3.612 |
| 56 | 6 | 2 | 62 | 0.0248 | 0.21156 | 1.0325 | 1.3742 | 1.2137 | 2.414 |
| 57 | 6 | 2 | 62 | 0.2463 | 0.34683 | 0.7501 | 1.8954 | 1.9982 | 3.708 |
| 58 | 6 | 2 | 62 | 0.121 | 0.31302 | 0.9866 | 1.88 | 1.6855 | 3.784 |
| 59 | 6 | 2 | 62 | 0.1411 | 0.32029 | 1.4257 | 1.2323 | 2.3573 | 3.258 |
| 60 | 6 | 2 | 62 | 0.3304 | 0.39557 | 1.4907 | 1.5976 | 2.2123 | 3.248 |
| 61 | 7 | 1 | 71 | 0.28282 | 1.2432 | 2.5351 | 2.14505 | 3.3618 | 3.88 |
| 62 | 7 | 1 | 71 | 0.27244 | 0.88614 | 0.3145 | 2.2433 | 4.403 | 4.328 |
| 63 | 7 | 1 | 71 | 0.16363 | 0.47079 | 1.4472 | 1.17268 | 3.202 | 2.872 |
| 64 | 7 | 1 | 71 | 0.38167 | 0.68246 | 0.5767 | 0.87668 | 2.098 | 2.464 |
| 65 | 7 | 1 | 71 | 0.11433 | 0.46703 | 1.5734 | 0.74528 | 2.1239 | 1.744 |
| 66 | 7 | 2 | 72 | 0.1361 | 0.14568 | 1.4245 | 0.2692 | 1.5885 | 1.714 |
| 67 | 7 | 2 | 72 | 0.0493 | 0.17821 | 1.5271 | 0.3482 | 1.5153 | 1.944 |
| 68 | 7 | 2 | 72 | 0.1342 | 0.28757 | 1.6359 | 0.3871 | 1.6587 | 1.268 |
| 69 | 7 | 2 | 72 | 0.2143 | 0.39016 | 1.5977 | 0.4298 | 1.7269 | 0.75 |
| 70 | 7 | 2 | 72 | 0.1724 | 0.25372 | 1.7595 | 0.6193 | 2.1687 | 2.04 |
| 71 | 8 | 1 | 81 | 0.14703 | 1.0595 | 1.3125 | 0.3293 | 2.5361 | 1.952 |
| 72 | 8 | 1 | 81 | 0.16362 | 0.74201 | 1.2457 | 0.5042 | 3.2689 | 1.666 |
| 73 | 8 | 1 | 81 | 0.11688 | 0.46815 | 0.8717 | 0.717 | 2.1094 | 2.138 |
| 74 | 8 | 1 | 81 | 0.05213 | 0.48752 | 1.1258 | 0.736 | 2.7041 | 1.614 |
| 75 | 8 | 1 | 81 | 0.0941 | 0.86669 | 0.8153 | 0.6032 | 2.9478 | 2.27 |
| 76 | 8 | 2 | 82 | 0.0587 | 0.35675 | 0.6915 | 0.5781 | 1.671 | 2.862 |
| 77 | 8 | 2 | 82 | 0.0099 | 0.17549 | 0.2032 | 0.3434 | 1.5395 | 3.114 |
| 78 | 8 | 2 | 82 | 0.1329 | 0.34021 | 0.6423 | 0.4058 | 1.2108 | 2.04 |
| 79 | 8 | 2 | 82 | 0.099 | 0.43462 | 0.958 | 0.2912 | 1.4518 | 2.324 |
| 80 | 8 | 2 | 82 | 0.0963 | 0.43616 | 0.2912 | 0.4908 | 0.8127 | 1.328 |
| 81 | 9 | 1 | 91 | 0.15812 | 0.54742 | 2.1628 | 1.1595 | 3.9466 | 3.236 |
| 82 | 9 | 1 | 91 | 0.12516 | 0.60997 | 1.6619 | 0.642 | 4.1546 | 2.996 |
| 83 | 9 | 1 | 91 | 0.04891 | 0.35732 | 2.0629 | 0.4071 | 3.5937 | 3.56 |
| 84 | 9 | 1 | 91 | 0.08702 | 0.73538 | 2.5066 | 0.3848 | 4.007 | 3.002 |
| 85 | 9 | 1 | 91 | 0.14947 | 0.7807 | 1.8469 | 0.9442 | 4.0685 | 3.238 |
| 86 | 9 | 2 | 92 | 0.0965 | 0.40155 | 1.252 | 1.2071 | 2.158 | 2.766 |
| 87 | 9 | 2 | 92 | 0.139 | 0.87072 | 2.1023 | 1.6002 | 2.1301 | 2.128 |
| 88 | 9 | 2 | 92 | 0.0618 | 0.50396 | 1.1939 | 1.4958 | 2.2731 | 2.383 |
| 89 | 9 | 2 | 92 | 0.1923 | 0.50881 | 1.3819 | 1.4727 | 2.0193 | 2.026 |
| 90 | 9 | 2 | 92 | 0.2555 | 0.55668 | 1.1741 | 1.163 | 2.4537 | 1.868 |
| 91 | 10 | 1 | 101 | 0.22352 | 0.51604 | 2.3681 | 1.7768 | 4.2277 | 2.634 |
| 92 | 10 | 1 | 101 | 0.22322 | 0.35701 | 2.9754 | 1.8457 | 3.6895 | 3.508 |
| 93 | 10 | 1 | 101 | 0.26499 | 0.51758 | 2.4417 | 1.5132 | 3.4743 | 2.44 |
| 94 | 10 | 1 | 101 | 0.29092 | 0.65401 | 2.5695 | 2.6676 | 3.7602 | 4.228 |
| 95 | 10 | 1 | 101 | 0.28023 | 0.83243 | 2.3723 | 2.3714 | 2.7236 | 3.582 |
| 96 | 10 | 2 | 102 | 0.0708 | 0.25024 | 1.931 | 0.4527 | 2.2044 | 2.124 |
| 97 | 10 | 2 | 102 | 0.1525 | 0.33786 | 1.8928 | 0.7854 | 2.8278 | 3.448 |
| 98 | 10 | 2 | 102 | 0.2027 | 0.3319 | 2.5024 | 1.4912 | 2.7855 | 2.844 |
| 99 | 10 | 2 | 102 | 0.1089 | 0.28309 | 2.0448 | 0.7623 | 2.5545 | 2.22 |
| 100 | 10 | 2 | 102 | 0.1271 | 0.39544 | 2.4202 | 0.4083 | 2.716 | 1.734 |
| 101 | 11 | 1 | 111 | 0.52587 | 1.11622 | 2.2371 | 1.3705 | 3.3116 | 4.008 |
| 102 | 11 | 1 | 111 | 0.32853 | 1.16718 | 2.2549 | 1.079 | 3.7471 | 3.168 |
| 103 | 11 | 1 | 111 | 0.26452 | 0.632 | 1.4662 | 1.0229 | 2.6316 | 3.904 |
| 104 | 11 | 1 | 111 | 0.24936 | 0.72698 | 1.7217 | 1.543 | 2.792 | 3.602 |
| 105 | 11 | 1 | 111 | 0.28194 | 0.49697 | 1.8774 | 1.4809 | 2.7956 | 3.666 |
| 106 | 11 | 2 | 112 | 0.1738 | 0.38593 | 2.4294 | 0.64356 | 2.7697 | 2.668 |
| 107 | 11 | 2 | 112 | 0.138 | 0.38623 | 1.6516 | 0.86051 | 1.2652 | 2.014 |
| 108 | 11 | 2 | 112 | 0.1294 | 0.227 | 0.8204 | 1.23847 | 1.3142 | 2.402 |
| 109 | 11 | 2 | 112 | 0.0952 | 0.36957 | 0.9775 | 1.44354 | 2.447 | 3.424 |
| 110 | 11 | 2 | 112 | 0.0111 | 0.52865 | 0.6695 | 0.73847 | 0.7355 | 3.148 |
| 111 | 12 | 1 | 121 | 0.24875 | 0.8176 | 1.9953 | 1.00274 | 2.5283 | 2.706 |
| 112 | 12 | 1 | 121 | 0.48654 | 0.38975 | 2.1136 | 1.23151 | 2.4805 | 2.26 |
| 113 | 12 | 1 | 121 | 0.36451 | 0.51184 | 1.5327 | 1.3523 | 2.4479 | 2.128 |
| 114 | 12 | 1 | 121 | 0.31374 | 0.56957 | 2.1877 | 1.36384 | 2.5942 | 3.418 |
| 115 | 12 | 1 | 121 | 0.59937 | 0.73823 | 2.5366 | 1.53194 | 3.0126 | 3.512 |
| 116 | 12 | 2 | 122 | 0.0247 | 0.20954 | 1.0407 | 0.9549 | 0.8769 | 1.808 |
| 117 | 12 | 2 | 122 | 0.1442 | 0.51855 | 1.9435 | 0.8222 | 1.7537 | 1.85 |
| 118 | 12 | 2 | 122 | 0.2071 | 0.44095 | 1.9196 | 0.7208 | 1.3239 | 1.702 |
| 119 | 12 | 2 | 122 | 0.2039 | 0.30585 | 2.1804 | 0.5537 | 1.027 | 1.476 |
| 120 | 12 | 2 | 122 | 0.2183 | 0.31521 | 1.9812 | 0.5738 | 0.7705 | 0.84 |
| 121 | 13 | 1 | 131 | 0.10625 | 0.27226 | 1.8037 | 1.0358 | 2.6322 | 3.302 |
| 122 | 13 | 1 | 131 | 0.07464 | 0.43811 | 1.8629 | 0.7423 | 2.359 | 3.642 |
| 123 | 13 | 1 | 131 | 0.23158 | 0.47495 | 2.1723 | 0.9543 | 3.5391 | 2.886 |
| 124 | 13 | 1 | 131 | 0.10441 | 0.26195 | 1.7951 | 0.8198 | 2.2598 | 2.048 |
| 125 | 13 | 1 | 131 | 0.19062 | 0.52866 | 1.5359 | 0.8397 | 2.94 | 2.842 |
| 126 | 13 | 2 | 132 | 0.1573 | 0.316 | 0.9854 | 1.1198 | 1.6704 | 1.806 |
| 127 | 13 | 2 | 132 | 0.3384 | 0.49338 | 1.804 | 1.0148 | 1.4658 | 1.31 |
| 128 | 13 | 2 | 132 | 0.2933 | 0.96314 | 1.9209 | 1.5825 | 1.4995 | 2.078 |
| 129 | 13 | 2 | 132 | 0.1782 | 0.524 | 1.4769 | 1.4908 | 1.856 | 1.908 |
| 130 | 13 | 2 | 132 | 0.2323 | 0.74749 | 2.0089 | 1.76843 | 1.9737 | 2.246 |
| 131 | 14 | 1 | 141 | 0.25064 | 0.86725 | 1.9759 | 1.9921 | 3.453 | 2.478 |
| 132 | 14 | 1 | 141 | 0.31332 | 0.42756 | 1.7452 | 0.798 | 2.932 | 2.506 |
| 133 | 14 | 1 | 141 | 0.13561 | 0.45442 | 1.8696 | 0.6627 | 2.7729 | 2.162 |
| 134 | 14 | 1 | 141 | 0.20601 | 0.44102 | 2.2244 | 0.9729 | 2.9449 | 2.968 |
| 135 | 14 | 1 | 141 | 0.22467 | 0.56869 | 2.0076 | 0.4766 | 2.8698 | 2.67 |
| 136 | 14 | 2 | 142 | 0.1922 | 1.05929 | 1.1418 | 0.3743 | 2.3951 | 2.401 |
| 137 | 14 | 2 | 142 | 0.2435 | 0.86763 | 1.2692 | 0.3319 | 2.0792 | 1.792 |
| 138 | 14 | 2 | 142 | 0.2451 | 0.71071 | 1.6218 | 0.51 | 2.4253 | 2.861 |
| 139 | 14 | 2 | 142 | 0.2433 | 0.43205 | 1.4697 | 1.103 | 1.8348 | 2.664 |
| 140 | 14 | 2 | 142 | 0.4431 | 0.70824 | 1.658 | 0.637 | 1.8185 | 2.094 |
| 141 | 15 | 1 | 151 | 0.39613 | 1.00897 | 2.8542 | 1.39041 | 3.6155 | 2.776 |
| 142 | 15 | 1 | 151 | 0.30424 | 0.63645 | 2.037 | 1.20354 | 3.4685 | 1.58 |
| 143 | 15 | 1 | 151 | 0.43444 | 0.39507 | 2.3303 | 1.9986 | 2.7918 | 3.236 |
| 144 | 15 | 1 | 151 | 0.4732 | 0.45276 | 2.6901 | 1.38933 | 4.2389 | 3.038 |
| 145 | 15 | 1 | 151 | 0.41037 | 0.42932 | 1.8763 | 1.40928 | 4.1386 | 3.516 |
| 146 | 15 | 2 | 152 | 0.2371 | 0.52726 | 1.7129 | 1.73131 | 1.5279 | 2.441 |
| 147 | 15 | 2 | 152 | 0.3709 | 0.59338 | 3.0598 | 1.00762 | 2.2027 | 2.97 |
| 148 | 15 | 2 | 152 | 0.3596 | 0.66573 | 2.9156 | 1.33276 | 2.2827 | 2.708 |
| 149 | 15 | 2 | 152 | 0.3093 | 0.75787 | 2.9483 | 1.46492 | 1.8217 | 2.269 |
| 150 | 15 | 2 | 152 | 0.3432 | 0.71463 | 2.4673 | 1.02786 | 2.1345 | 2.474 |
| 151 | 16 | 1 | 161 | 0.4016 | 0.40666 | 2.5171 | 2.9918 | 3.701 | 2.911 |
| 152 | 16 | 1 | 161 | 0.5153 | 0.48126 | 2.5365 | 2.5304 | 3.7866 | 2.511 |
| 153 | 16 | 1 | 161 | 0.3175 | 0.57554 | 2.5999 | 2.4419 | 3.89 | 3.051 |
| 154 | 16 | 1 | 161 | 0.3124 | 0.47511 | 2.2762 | 1.9558 | 3.8519 | 2.272 |
| 155 | 16 | 1 | 161 | 0.4096 | 0.27637 | 2.1041 | 1.5918 | 3.6215 | 3.648 |
| 156 | 16 | 2 | 162 | 0.2302 | 0.6817 | 2.689 | 1.77568 | 2.0447 | NA |
| 157 | 16 | 2 | 162 | 0.0643 | 0.63363 | 1.6797 | 2.11901 | 1.5684 | NA |
| 158 | 16 | 2 | 162 | 0.1337 | 0.43665 | 1.1733 | 1.41315 | 1.6061 | NA |
| 159 | 16 | 2 | 162 | 0.2718 | 0.58392 | 1.4227 | 1.39839 | 1.705 | NA |
| 160 | 16 | 2 | 162 | 0.1843 | 0.73587 | 2.002 | 2.49363 | 1.5975 | NA |
| 161 | 17 | 1 | 171 | 0.2715 | 0.386 | 1.1367 | 0.8594 | 2.4641 | 2.77 |
| 162 | 17 | 1 | 171 | 0.2289 | 0.28115 | 1.1589 | 0.9652 | 2.1153 | 2.81 |
| 163 | 17 | 1 | 171 | 0.3162 | 0.5492 | 1.1793 | 1.3495 | 2.0881 | 3.32 |
| 164 | 17 | 1 | 171 | 0.427 | 0.62663 | 1.8293 | 1.1044 | 3.2304 | 3.115 |
| 165 | 17 | 1 | 171 | 0.3589 | 0.34479 | 1.4607 | 1.2604 | 2.7661 | 3.065 |
| 166 | 17 | 2 | 172 | 0.1495 | 0.92721 | 2.5912 | 1.59571 | 2.7007 | 2.042 |
| 167 | 17 | 2 | 172 | 0.0944 | 0.61716 | 1.1156 | 0.31718 | 1.3655 | 1.808 |
| 168 | 17 | 2 | 172 | 0.1104 | 0.96358 | 1.4221 | 1.03399 | 2.4474 | 1.208 |
| 169 | 17 | 2 | 172 | 0.2875 | 1.08856 | 1.3543 | 1.53097 | 1.862 | 2.392 |
| 170 | 17 | 2 | 172 | 0.09 | 1.30978 | 1.5584 | 0.89513 | 1.9976 | 1.772 |
| 171 | 18 | 1 | 181 | 0.3667 | 0.47423 | 1.4033 | 1.1763 | 2.9837 | 2.248 |
| 172 | 18 | 1 | 181 | 0.3956 | 0.31417 | 1.489 | 1.3988 | 2.4805 | 1.922 |
| 173 | 18 | 1 | 181 | 0.4184 | 0.41057 | 2.0706 | 0.9826 | 3.2175 | 1.872 |
| 174 | 18 | 1 | 181 | 0.3687 | 0.58541 | 1.5934 | 1.7718 | 3.0742 | 2.756 |
| 175 | 18 | 1 | 181 | 0.2745 | 0.22785 | 1.7582 | 1.1589 | 3.1316 | 3.468 |
| 176 | 18 | 2 | 182 | 0.1477 | 0.35094 | 1.5587 | 1.0134 | 2.2997 | 2.531 |
| 177 | 18 | 2 | 182 | 0.1933 | 0.27972 | 2.1244 | 0.9613 | 2.1322 | 2.921 |
| 178 | 18 | 2 | 182 | 0.1625 | 0.41609 | 1.868 | 1.0937 | 2.5886 | 2.702 |
| 179 | 18 | 2 | 182 | 0.1649 | 0.34767 | 1.8009 | 0.8081 | 2.4433 | 2.708 |
| 180 | 18 | 2 | 182 | 0.1065 | 0.26751 | 1.5448 | 0.9981 | 2.1647 | 2.572 |
| 181 | 19 | 1 | 191 | 0.3524 | 0.35305 | 1.4013 | 0.9729 | 0.8762 | 1.886 |
| 182 | 19 | 1 | 191 | 0.3986 | 0.36525 | 1.2698 | 0.4545 | 0.6341 | 1.182 |
| 183 | 19 | 1 | 191 | 0.28 | 0.28356 | 1.5763 | 0.6673 | 0.934 | 1.332 |
| 184 | 19 | 1 | 191 | 0.2886 | 0.28824 | 1.386 | 0.6311 | 1.166 | 1.516 |
| 185 | 19 | 1 | 191 | 0.1436 | 0.11665 | 0.8306 | 0.3844 | 1.3547 | 1.896 |
| 186 | 19 | 2 | 192 | 0.1657 | 0.59722 | 0.4641 | 0.3293 | 0.2724 | 0.929 |
| 187 | 19 | 2 | 192 | 0.1525 | 0.25998 | 0.484 | 0.2791 | 0.6494 | 1 |
| 188 | 19 | 2 | 192 | 0.0668 | 0.20478 | 0.392 | 0.2269 | 0.2054 | 1.032 |
| 189 | 19 | 2 | 192 | 0.0824 | 0.27228 | 0.6791 | 0.2658 | 0.1895 | 0.828 |
| 190 | 19 | 2 | 192 | 0.0803 | 0.18298 | 0.6967 | 0.4149 | 0.1152 | 0.661 |
| 191 | 20 | 1 | 201 | 0.0719 | 0.09796 | 1.1237 | 0.8807 | 2.1176 | 2.716 |
| 192 | 20 | 1 | 201 | 0.167 | 0.35299 | 1.8701 | 1.3574 | 3.144 | 3.02 |
| 193 | 20 | 1 | 201 | 0.0897 | 0.21269 | 1.0109 | 1.0922 | 2.1411 | 1.398 |
| 194 | 20 | 1 | 201 | 0.0801 | 0.2099 | 1.1497 | 1.4263 | 1.8814 | 1.894 |
| 195 | 20 | 1 | 201 | 0.1332 | 0.73676 | 2.0073 | 1.5203 | 2.7864 | 2.445 |
| 196 | 20 | 2 | 202 | 0.0801 | 0.29738 | 1.5112 | 0.5185 | 2.7014 | 3.068 |
| 197 | 20 | 2 | 202 | 0.0568 | 0.48249 | 1.4387 | 0.327 | 1.3351 | 2.088 |
| 198 | 20 | 2 | 202 | 0.0401 | 0.27137 | 1.4388 | 0.5843 | 1.5695 | 2.402 |
| 199 | 20 | 2 | 202 | 0.0563 | 0.4668 | 1.5447 | 0.5574 | 2.0557 | 2.091 |
| 200 | 20 | 2 | 202 | 0.082 | 0.58909 | 1.8503 | 1.0107 | 2.1563 | 2.228 |
| 201 | 21 | 1 | 211 | 0.0652 | 0.39055 | 1.7958 | 1.436 | NA | NA |
| 202 | 21 | 1 | 211 | 0.1133 | 0.59702 | 1.3388 | 1.1379 | 3.4465 | 1.972 |
| 203 | 21 | 1 | 211 | 0.0863 | 0.2687 | 1.6285 | 1.1551 | 2.7065 | NA |
| 204 | 21 | 1 | 211 | 0.136 | 0.23339 | 1.5639 | 1.558 | 3.2835 | 2.312 |
| 205 | 21 | 1 | 211 | 0.1338 | 0.3667 | 1.1331 | 1.0774 | 1.8941 | 1.862 |
| 206 | 21 | 2 | 212 | 0.1 | 0.16815 | 1.9002 | 1.5512 | 1.2494 | 2.438 |
| 207 | 21 | 2 | 212 | 0.1376 | 0.2928 | 1.5392 | 1.267 | 1.3615 | 2.434 |
| 208 | 21 | 2 | 212 | 0.0954 | 0.43814 | 0.8952 | 1.262 | 1.5989 | 1.83 |
| 209 | 21 | 2 | 212 | 0.2309 | 0.51159 | 1.6372 | 1.5202 | 1.1917 | 1.868 |
| 210 | 21 | 2 | 212 | 0.1109 | 0.41393 | 1.3872 | 2.1242 | 1.1703 | 3.098 |
| 211 | 22 | 1 | 221 | 0.1297 | 0.27026 | 0.9652 | 0.7838 | 1.4964 | 2.06 |
| 212 | 22 | 1 | 221 | 0.2375 | 0.35486 | 1.7626 | 0.9308 | 1.8797 | 2.1 |
| 213 | 22 | 1 | 221 | 0.1533 | 0.25571 | 1.2495 | 0.8384 | 1.8143 | 2.174 |
| 214 | 22 | 1 | 221 | 0.2066 | 0.34021 | 0.9925 | 0.9506 | 2.0076 | 2.272 |
| 215 | 22 | 1 | 221 | 0.2063 | 0.40745 | 1.1396 | 0.7827 | 1.8228 | 1.646 |
| 216 | 22 | 2 | 222 | 0.2342 | 1.22633 | 1.647 | 1.6842 | 2.5422 | 2.884 |
| 217 | 22 | 2 | 222 | 0.3275 | 1.19001 | 1.9527 | 1.8224 | 3.1686 | 2.788 |
| 218 | 22 | 2 | 222 | 0.2396 | 0.99972 | 1.4432 | 1.212 | 2.2716 | 2.182 |
| 219 | 22 | 2 | 222 | 0.1097 | 0.77035 | 1.6213 | 0.7179 | 2.0447 | 1.988 |
| 220 | 22 | 2 | 222 | 0.1536 | 0.71922 | 1.4335 | 1.2232 | 2.0137 | 2.974 |
| 221 | 23 | 1 | 231 | 0.0641 | 0.35784 | 1.024 | 1.03 | 1.3918 | 3.308 |
| 222 | 23 | 1 | 231 | 0.0572 | 0.20118 | 1.1307 | 1.1446 | 2.1897 | 2.616 |
| 223 | 23 | 1 | 231 | 0.0518 | 0.09331 | 0.5528 | 0.4414 | 2.224 | 2.888 |
| 224 | 23 | 1 | 231 | 0.0788 | 0.11927 | 0.4725 | 1.0303 | 2.0399 | 2.09 |
| 225 | 23 | 1 | 231 | 0.0973 | 0.24401 | 0.9023 | 0.5939 | 1.9521 | 1.296 |
| 226 | 23 | 2 | 232 | 0.137 | 0.2223 | 1.0472 | 0.3792 | 1.3135 | 1.888 |
| 227 | 23 | 2 | 232 | 0.0467 | 0.20118 | 0.5718 | 0.2745 | 0.8806 | 2.238 |
| 228 | 23 | 2 | 232 | 0.0985 | 0.29135 | 0.4538 | 0.2749 | 1.0992 | 2.61 |
| 229 | 23 | 2 | 232 | 0.1491 | 0.35899 | 0.9852 | 0.357 | 1.5702 | 3.368 |
| 230 | 23 | 2 | 232 | 0.1579 | 0.36812 | 0.9041 | 0.4045 | 1.3715 | 3.18 |
| 231 | 24 | 1 | 241 | 0.3775 | 0.79217 | 2.0137 | 2.68347 | 2.923 | NA |
| 232 | 24 | 1 | 241 | 0.3175 | 0.8973 | 2.0129 | 4.46412 | 2.726 | NA |
| 233 | 24 | 1 | 241 | 0.5059 | 0.49151 | 2.1345 | 2.57121 | 4.0023 | NA |
| 234 | 24 | 1 | 241 | 0.481 | 0.75245 | 1.9088 | 2.47008 | 3.1204 | NA |
| 235 | 24 | 1 | 241 | 0.4753 | 1.25434 | 2.7903 | 3.3674 | 2.6218 | NA |
| 236 | 24 | 2 | 242 | 0.1075 | 0.18029 | 0.4715 | 0.7264 | 1.0229 | 0.912 |
| 237 | 24 | 2 | 242 | 0.3115 | 0.40109 | 1.0004 | 0.78 | 1.259 | 2.172 |
| 238 | 24 | 2 | 242 | 0.3321 | 0.43769 | 0.8437 | 1.0991 | 1.246 | 2.17 |
| 239 | 24 | 2 | 242 | 0.3282 | 0.53219 | 0.8601 | 1.0273 | 1.3159 | 0.692 |
| 240 | 24 | 2 | 242 | 0.782 | 0.64539 | 1.3177 | 2.1492 | 2.1269 | 1.002 |
| 241 | 25 | 1 | 251 | 0.3775 | 0.69857 | 1.8118 | 2.9635 | 2.3745 | 3.54 |
| 242 | 25 | 1 | 251 | 0.3175 | 0.95672 | 1.5296 | 2.5138 | 3.4356 | 3.322 |
| 243 | 25 | 1 | 251 | 0.5059 | 0.45511 | 0.8988 | 1.4651 | 2.599 | 3.306 |
| 244 | 25 | 1 | 251 | 0.481 | 0.59713 | 0.873 | 1.7128 | 3.3052 | 3.318 |
| 245 | 25 | 1 | 251 | 0.4753 | 0.4756 | 1.0432 | 1.961 | 2.8868 | 2.354 |
| 246 | 25 | 2 | 252 | 0.1099 | 0.27143 | 0.5727 | 0.9325 | 1.5659 | 2.278 |
| 247 | 25 | 2 | 252 | 0.0658 | 0.26332 | 1.0107 | 0.9593 | 1.6354 | 2.112 |
| 248 | 25 | 2 | 252 | 0.2484 | 0.33329 | 1.1469 | 1.9827 | 1.8466 | 2.672 |
| 249 | 25 | 2 | 252 | 0.2473 | 0.14278 | 0.6091 | 1.5652 | 0.6044 | 2.328 |
| 250 | 25 | 2 | 252 | 0.1501 | 0.15558 | 0.5976 | 1.3751 | 1.3918 | 0.88 |
| 251 | 26 | 1 | 261 | 0.0798 | 0.1944 | 1.1938 | 0.5907 | 0.5272 | 0.998 |
| 252 | 26 | 1 | 261 | 0.2145 | 0.3194 | 1.3237 | 0.6246 | 0.2395 | 0.148 |
| 253 | 26 | 1 | 261 | 0.1319 | 0.58493 | 1.9109 | 0.5891 | 0.4115 | 0.158 |
| 254 | 26 | 1 | 261 | 0.0644 | 0.388 | 1.2004 | 0.6508 | 0.4898 | 1.15 |
| 255 | 26 | 1 | 261 | 0.1613 | 0.41816 | 1.3615 | 0.5083 | 0.1704 | 0.744 |
| 256 | 26 | 2 | 262 | 0.0084 | 0.16934 | 0.9364 | 0.6796 | 0.7714 | 0.982 |
| 257 | 26 | 2 | 262 | 0.0116 | 0.18782 | 0.3293 | 0.85 | 0.9378 | 0.042 |
| 258 | 26 | 2 | 262 | 0.0158 | 0.22044 | 0.9405 | 1.0896 | 1.001 | 0.072 |
| 259 | 26 | 2 | 262 | 0.0698 | 0.37876 | 0.5433 | 0.769 | 0.8223 | 0.988 |
| 260 | 26 | 2 | 262 | 0.0296 | 0.36622 | 0.8613 | 0.954 | 0.8522 | 0.778 |
| 261 | 27 | 1 | 271 | 0.1255 | 0.249 | 0.3457 | 0.2159 | 0.2373 | 0.46 |
| 262 | 27 | 1 | 271 | 0.0417 | 0.0259 | 0.0201 | 0.0389 | NA | 0.052 |
| 263 | 27 | 1 | 271 | 0.0691 | 0.40624 | 0.4135 | 0.2652 | 0.0818 | 0.418 |
| 264 | 27 | 1 | 271 | 0.0772 | 0.60312 | 1.0268 | 0.3363 | 0.1214 | 0.422 |
| 265 | 27 | 1 | 271 | 0.0643 | 0.1701 | 0.31 | 0.3532 | 0.0801 | 0.586 |
| 266 | 27 | 2 | 272 | 0.0188 | 0.15081 | 0.3678 | 0.0162 | 0.1175 | 0.102 |
| 267 | 27 | 2 | 272 | 0.0293 | 0.40295 | 0.4039 | 0.072 | 0.2793 | 0.028 |
| 268 | 27 | 2 | 272 | 0.1015 | 0.39901 | 0.2426 | 0.1386 | 0.0785 | 0.034 |
| 269 | 27 | 2 | 272 | 0.0932 | 0.18321 | 0.1452 | 0.0963 | 0.0805 | 0.03 |
| 270 | 27 | 2 | 272 | 0.0449 | 0.12879 | 0.2433 | 0.1617 | 0.2318 | 0.068 |
| 271 | 28 | 1 | 281 | 0.1661 | 0.99689 | 1.9715 | 2.2254 | 2.0934 | 2.236 |
| 272 | 28 | 1 | 281 | 0.2624 | 0.81971 | 2.1433 | 2.5457 | 2.1589 | 2.19 |
| 273 | 28 | 1 | 281 | 0.2217 | 0.63229 | 1.9912 | 1.957 | 2.104 | 2.446 |
| 274 | 28 | 1 | 281 | 0.313 | 0.77881 | 2.2843 | 2.836 | 1.8383 | 2.02 |
| 275 | 28 | 1 | 281 | 0.1736 | 0.19503 | 1.8492 | 2.2836 | 1.5537 | 2.272 |
| 276 | 28 | 2 | 282 | 0.1245 | 0.68778 | 0.7514 | 0.5917 | 1.4578 | 2.548 |
| 277 | 28 | 2 | 282 | 0.1766 | 0.72204 | 0.7721 | 0.5064 | 1.229 | 1.734 |
| 278 | 28 | 2 | 282 | 0.1325 | 0.46093 | 0.6593 | 0.5155 | 1.0892 | 2.004 |
| 279 | 28 | 2 | 282 | 0.1852 | 0.40688 | 0.447 | 0.4566 | 0.9922 | 2.018 |
| 280 | 28 | 2 | 282 | 0.0768 | 0.51807 | 0.5575 | 0.0976 | 1.1079 | 1.838 |
| 281 | 29 | 1 | 291 | 0.157 | 0.60694 | 1.9676 | 2.12078 | 2.3743 | 3.52 |
| 282 | 29 | 1 | 291 | 0.1528 | 1.04552 | 2.5179 | 2.2247 | 3.2892 | 3.57 |
| 283 | 29 | 1 | 291 | 0.1116 | 0.32025 | 1.7756 | 1.76002 | 2.711 | 3.462 |
| 284 | 29 | 1 | 291 | 0.0645 | 0.32871 | 1.1514 | 1.55928 | 2.9917 | 3.188 |
| 285 | 29 | 1 | 291 | 0.1878 | 0.67946 | 2.3695 | 2.17016 | 3.2462 | 3.782 |
| 286 | 29 | 2 | 292 | 0.1232 | 0.56502 | 1.5933 | 1.27673 | 0.8197 | 1.842 |
| 287 | 29 | 2 | 292 | 0.1037 | 0.56406 | 1.532 | 0.94714 | 1.5066 | 2.574 |
| 288 | 29 | 2 | 292 | 0.1048 | 0.36931 | 1.9933 | 0.87652 | 1.9748 | 2.928 |
| 289 | 29 | 2 | 292 | 0.1123 | 0.58967 | 2.0981 | 0.96254 | 2.284 | 3.414 |
| 290 | 29 | 2 | 292 | 0.1377 | 0.71676 | 2.804 | 1.06487 | 1.8391 | 2.878 |
| 291 | 30 | 1 | 301 | 0.5628 | 0.77858 | 1.985 | 1.3344 | 3.1314 | 3.488 |
| 292 | 30 | 1 | 301 | 0.4953 | 0.89033 | 0.7948 | 1.7976 | 1.9655 | 0.676 |
| 293 | 30 | 1 | 301 | 0.545 | 0.46863 | 1.9857 | 1.5138 | 3.2951 | 1.65 |
| 294 | 30 | 1 | 301 | 0.5544 | 0.72852 | 1.6853 | 1.6803 | 2.6919 | NA |
| 295 | 30 | 1 | 301 | 0.5091 | 0.39121 | 0.8051 | 1.3047 | 2.7481 | 0.938 |
| 296 | 30 | 2 | 302 | 0.2221 | 0.45315 | 1.2407 | 0.5786 | 1.7278 | 3.212 |
| 297 | 30 | 2 | 302 | 0.1692 | 0.35292 | 1.4932 | 0.6365 | 2.4104 | 2.71 |
| 298 | 30 | 2 | 302 | 0.1734 | 0.2761 | 1.1665 | 0.8052 | 2.1726 | 2.92 |
| 299 | 30 | 2 | 302 | 0.297 | 0.18512 | 1.3345 | 0.6104 | 1.7868 | 1.63 |
| 300 | 30 | 2 | 302 | 0.3448 | 0.33886 | 1.1157 | 1.4466 | 2.1726 | 3.084 |
| 301 | 31 | 1 | 311 | 0.0422 | 0.1765 | 0.1914 | 0.3656 | 0.045 | 0.368 |
| 302 | 31 | 1 | 311 | 0.1136 | 0.51333 | 1.3878 | 1.4064 | 0.2664 | 0.858 |
| 303 | 31 | 1 | 311 | 0.0638 | 0.51357 | 0.7949 | 1.3941 | 0.3006 | 0.624 |
| 304 | 31 | 1 | 311 | 0.0484 | 0.30062 | 1.1319 | 1.4555 | 0.2418 | 0.712 |
| 305 | 31 | 1 | 311 | 0.019 | 0.27895 | 0.7495 | 1.2634 | 0.1333 | 0.26 |
| 306 | 31 | 2 | 312 | 0.0575 | 0.28958 | 0.6948 | 0.5335 | 0.0965 | 0.241 |
| 307 | 31 | 2 | 312 | 0.0477 | 0.34077 | 0.9825 | 0.6493 | 0.2188 | 0.598 |
| 308 | 31 | 2 | 312 | 0.0446 | 0.41977 | 0.3643 | 0.4753 | 0.1713 | 0.282 |
| 309 | 31 | 2 | 312 | 0.0227 | 0.38916 | 0.4763 | 0.4521 | 0.1574 | 0.502 |
| 310 | 31 | 2 | 312 | 0.0437 | 0.41225 | 0.4421 | 0.6078 | 0.3896 | 0.784 |
| 311 | 32 | 1 | 321 | 0.3648 | 0.68273 | 0.516 | 1.6416 | 2.7641 | NA |
| 312 | 32 | 1 | 321 | 0.2987 | 0.5474 | 0.9 | 1.6948 | 2.8429 | NA |
| 313 | 32 | 1 | 321 | 0.3821 | 0.67987 | 1.5788 | 1.8318 | 3.1751 | NA |
| 314 | 32 | 1 | 321 | 0.2352 | 0.62824 | 1.256 | 1.4995 | 3.1804 | 2.29 |
| 315 | 32 | 1 | 321 | 0.193 | 0.37188 | 1.1911 | 1.5318 | 2.4708 | NA |
| 316 | 32 | 2 | 322 | 0.4022 | 0.98246 | 1.0089 | 1.7427 | 1.7697 | 3.018 |
| 317 | 32 | 2 | 322 | 0.3927 | 0.97815 | 0.9549 | 2.1442 | 1.4243 | 3.202 |
| 318 | 32 | 2 | 322 | 0.1493 | 0.36279 | 0.4921 | 1.1777 | 1.1124 | 2.152 |
| 319 | 32 | 2 | 322 | 0.2927 | 1.27968 | 0.3854 | 1.742 | 1.3363 | 2.948 |
| 320 | 32 | 2 | 322 | 0.2631 | 1.13225 | 1.4909 | 2.4097 | 2.1263 | 3.742 |
| 321 | 33 | 1 | 331 | 0.0281 | 0.25139 | 0.6316 | 0.7521 | 1.6732 | 1.618 |
| 322 | 33 | 1 | 331 | 0.0927 | 0.39732 | 1.8581 | 1.2195 | 2.1747 | 1.596 |
| 323 | 33 | 1 | 331 | 0.099 | 0.46502 | 1.4399 | 1.0105 | 1.8848 | 1.83 |
| 324 | 33 | 1 | 331 | 0.0652 | 0.28235 | 1.3343 | 1.2445 | 2.3046 | 1.752 |
| 325 | 33 | 1 | 331 | 0.2092 | 0.48931 | 1.4912 | 1.2775 | 2.1003 | 1.892 |
| 326 | 33 | 2 | 332 | 0.1767 | 0.20312 | 1.0578 | 0.2335 | 1.3937 | 1.261 |
| 327 | 33 | 2 | 332 | 0.0846 | 0.31172 | 0.9564 | 0.36 | 1.4763 | 0.861 |
| 328 | 33 | 2 | 332 | 0.08 | 0.24491 | 0.5547 | 0.0918 | 0.9412 | 0.538 |
| 329 | 33 | 2 | 332 | 0.0775 | 0.14383 | 0.7331 | 0.304 | 0.71 | 0.698 |
| 330 | 33 | 2 | 332 | 0.0847 | 0.32792 | 0.5287 | 0.3443 | 0.9458 | 0.834 |
| 331 | 34 | 1 | 341 | 0.3009 | 1.24806 | 1.833 | 2.4644 | 3.3757 | 2.754 |
| 332 | 34 | 1 | 341 | 0.3586 | 0.80121 | 2.1081 | 2.2449 | 3.845 | 4.132 |
| 333 | 34 | 1 | 341 | 0.2249 | 0.49848 | 1.7546 | 1.8542 | 4.4186 | 4.656 |
| 334 | 34 | 1 | 341 | 0.2377 | 0.80807 | 2.276 | 0.5726 | 1.2007 | NA |
| 335 | 34 | 1 | 341 | 0.3166 | 0.97892 | 2.138 | 2.3324 | 3.7453 | 3.178 |
| 336 | 34 | 2 | 342 | 0.059 | 0.81992 | 1.2654 | 1.0749 | 1.4856 | 3.204 |
| 337 | 34 | 2 | 342 | 0.301 | 0.77193 | 1.6256 | 0.6846 | 1.0872 | 3.654 |
| 338 | 34 | 2 | 342 | 0.1515 | 0.72423 | 2.2252 | 0.8968 | 1.8436 | 1.694 |
| 339 | 34 | 2 | 342 | 0.1051 | 0.95193 | 1.2789 | 0.785 | 1.6902 | 2.842 |
| 340 | 34 | 2 | 342 | 0.1465 | 0.67062 | 0.913 | 0.757 | 1.4535 | 2.854 |
| 341 | 35 | 1 | 351 | 0.1937 | 0.4647 | 1.5608 | 2.0081 | 3.0414 | 3.848 |
| 342 | 35 | 1 | 351 | 0.3213 | 0.81967 | 1.1644 | 2.2422 | 3.7258 | 4.218 |
| 343 | 35 | 1 | 351 | 0.244 | 0.4411 | 1.3108 | 2.4816 | 2.7196 | 3.94 |
| 344 | 35 | 1 | 351 | 0.3323 | 0.76849 | 2.279 | 1.8542 | 0.8851 | 1.534 |
| 345 | 35 | 1 | 351 | 0.3148 | 0.85862 | 2.4898 | 2.7261 | 2.566 | 4.82 |
| 346 | 35 | 2 | 352 | 0.1243 | 0.41236 | 0.5596 | 0.7313 | 1.9387 | 1.5 |
| 347 | 35 | 2 | 352 | 0.0445 | 0.65874 | 1.3399 | 0.7151 | 2.0632 | 2.782 |
| 348 | 35 | 2 | 352 | 0.0549 | 1.07814 | 2.0285 | 0.5744 | 2.974 | 2.608 |
| 349 | 35 | 2 | 352 | 0.0798 | 0.67112 | 1.46 | 0.7551 | 1.6911 | 2.328 |
| 350 | 35 | 2 | 352 | 0.1273 | 0.90593 | 1.3806 | 1.0228 | 1.4603 | 3.088 |
| 351 | 36 | 1 | 361 | 0.3565 | 0.57325 | 1.6315 | 2.4527 | 3.8701 | 4.7 |
| 352 | 36 | 1 | 361 | 0.4323 | 0.51682 | 1.6868 | 1.5002 | 2.9346 | 3.396 |
| 353 | 36 | 1 | 361 | 0.2812 | 0.8673 | 1.9272 | 0.9013 | 2.9706 | 3.052 |
| 354 | 36 | 1 | 361 | 0.3291 | 0.52586 | 1.4464 | 1.0246 | 3.0964 | 3.086 |
| 355 | 36 | 1 | 361 | 0.3617 | 0.88391 | 2.2925 | 1.2688 | 4.1364 | 3.426 |
| 356 | 36 | 2 | 362 | 0.537 | 0.52874 | 1.4197 | 0.476 | 1.312 | 1.791 |
| 357 | 36 | 2 | 362 | 0.267 | 0.51449 | 1.1791 | 1.0134 | 1.5894 | 2.288 |
| 358 | 36 | 2 | 362 | 0.1961 | 0.44462 | 1.1776 | 0.4202 | 2.0618 | 2.591 |
| 359 | 36 | 2 | 362 | 0.2018 | 0.41047 | 1.3011 | 0.3131 | 1.8002 | 2.608 |
| 360 | 36 | 2 | 362 | 0.4134 | 0.58363 | 1.515 | 0.5714 | 2.4647 | 2.838 |
| 361 | 37 | 1 | 371 | 0.3136 | 0.58559 | 1.5227 | 2.2069 | 2.9681 | 3.588 |
| 362 | 37 | 1 | 371 | 0.3466 | 0.59839 | 2.0663 | 1.9511 | 3.2889 | 3.038 |
| 363 | 37 | 1 | 371 | 0.2606 | 0.44129 | 0.9411 | 0.8753 | 2.9023 | 3.502 |
| 364 | 37 | 1 | 371 | 0.2577 | 0.42432 | 1.4343 | 1.6109 | 2.6729 | 4.372 |
| 365 | 37 | 1 | 371 | 0.4207 | 0.4076 | 1.3357 | 1.1453 | 4.078 | 2.538 |
| 366 | 37 | 2 | 372 | 0.329 | 0.70877 | 0.972 | 0.5294 | 2.4595 | 3.278 |
| 367 | 37 | 2 | 372 | 0.1346 | 0.67172 | 1.3141 | 0.6587 | 2.3084 | 2.398 |
| 368 | 37 | 2 | 372 | 0.1532 | 0.74579 | 1.3882 | 1.0472 | 2.4439 | 2.928 |
| 369 | 37 | 2 | 372 | 0.0501 | 0.71677 | 1.5461 | 0.7742 | 3.1363 | 2.71 |
| 370 | 37 | 2 | 372 | 0.0713 | 0.51561 | 1.5581 | 1.2759 | 2.1277 | 3.44 |
| 371 | 38 | 1 | 381 | 0.2424 | 0.44683 | 1.9474 | 1.3152 | 2.7567 | 3.424 |
| 372 | 38 | 1 | 381 | 0.325 | 0.52991 | 1.8544 | 0.9693 | 2.1077 | 2.93 |
| 373 | 38 | 1 | 381 | 0.3282 | 0.57578 | 1.8004 | 1.1757 | 3.0161 | 3.144 |
| 374 | 38 | 1 | 381 | 0.1664 | 0.45059 | 1.7892 | 1.2954 | 2.9802 | 3.084 |
| 375 | 38 | 1 | 381 | 0.2416 | 0.61534 | 1.6863 | 1.4065 | 2.6802 | 3.646 |
| 376 | 38 | 2 | 382 | 0.0817 | 0.52392 | 0.9092 | 0.1778 | 1.7417 | 3.2 |
| 377 | 38 | 2 | 382 | 0.0943 | 0.27636 | 0.9085 | 1.2657 | 0.9954 | 2.934 |
| 378 | 38 | 2 | 382 | 0.0492 | 0.18349 | 0.6232 | 1.086 | 1.4669 | 2.404 |
| 379 | 38 | 2 | 382 | 0.1147 | 0.44523 | 0.8256 | 1.0109 | 1.3345 | 2.148 |
| 380 | 38 | 2 | 382 | 0.277 | 0.64648 | 1.6159 | 3.0201 | 1.278 | 3.286 |
| 381 | 39 | 1 | 391 | 0.3131 | 0.38979 | 1.5295 | 2.7985 | 2.9953 | 3.945 |
| 382 | 39 | 1 | 391 | 0.2194 | 0.5815 | 1.2734 | 2.4916 | 3.1291 | 3.435 |
| 383 | 39 | 1 | 391 | 0.2067 | 0.68783 | 1.2324 | 2.6869 | 3.3306 | 3.565 |
| 384 | 39 | 1 | 391 | 0.163 | 0.78024 | 1.114 | 1.8365 | 1.1601 | 2.085 |
| 385 | 39 | 1 | 391 | 0.1467 | 0.81355 | 1.3398 | 2.0529 | 3.613 | 3.725 |
| 386 | 39 | 2 | 392 | 0.2246 | 0.44193 | 1.5215 | 1.6992 | 2.3527 | 2.468 |
| 387 | 39 | 2 | 392 | 0.1253 | 0.56975 | 1.2023 | 2.3754 | 2.1272 | 3.728 |
| 388 | 39 | 2 | 392 | 0.2711 | 0.65095 | 1.6628 | 2.3669 | 2.0568 | 2.412 |
| 389 | 39 | 2 | 392 | 0.176 | 0.55028 | 1.0024 | 1.7944 | 2.2681 | 2.728 |
| 390 | 39 | 2 | 392 | 0.1174 | 0.56431 | 1.0961 | 1.2873 | 2.2352 | 3.942 |
| 391 | 40 | 1 | 401 | 0.169 | 1.00142 | 1.9653 | 1.8341 | 2.4829 | 2.895 |
| 392 | 40 | 1 | 401 | 0.2115 | 1.28692 | 2.0375 | 2.4463 | 2.5103 | 2.554 |
| 393 | 40 | 1 | 401 | 0.1611 | 0.95288 | 1.8616 | 1.9589 | 1.8546 | 2.23 |
| 394 | 40 | 1 | 401 | 0.1878 | 0.65959 | 1.6939 | 2.1523 | 2.3914 | 2.6 |
| 395 | 40 | 1 | 401 | 0.082 | 0.26821 | 1.355 | 1.1759 | 1.3534 | 2.116 |
| 396 | 40 | 2 | 402 | 0.4825 | 0.46897 | 2.2775 | 1.1867 | 1.9937 | 2.832 |
| 397 | 40 | 2 | 402 | 0.3139 | 0.51962 | 1.8999 | 1.2294 | 2.3687 | 2.3 |
| 398 | 40 | 2 | 402 | 0.3224 | 0.67519 | 1.8854 | 0.6731 | 1.8372 | 2.998 |
| 399 | 40 | 2 | 402 | 0.2426 | 0.43405 | 1.5663 | 0.7854 | 2.2878 | 2.518 |
| 400 | 40 | 2 | 402 | 0.2958 | 0.50664 | 1.8226 | 0.5989 | 2.0869 | 2.318 |
| 401 | 41 | 1 | 411 | 0.3345 | 0.52192 | 1.5096 | 2.3973 | 3.0066 | 4.104 |
| 402 | 41 | 1 | 411 | 0.2441 | 0.25158 | 1.6291 | 1.4968 | 3.733 | 3.366 |
| 403 | 41 | 1 | 411 | 0.2783 | 0.37849 | 1.3845 | 1.4478 | 3.3456 | 3.54 |
| 404 | 41 | 1 | 411 | 0.2767 | 0.52684 | 1.4348 | 1.9817 | 3.3354 | 3.77 |
| 405 | 41 | 1 | 411 | 0.3884 | 0.49586 | 1.2692 | 1.7475 | 3.7781 | 4.04 |
| 406 | 41 | 2 | 412 | 0.3198 | 0.50142 | 1.4276 | 1.3187 | 3.5185 | 3.33 |
| 407 | 41 | 2 | 412 | 0.4165 | 0.44396 | 2.1676 | 0.7902 | 2.0667 | 2.328 |
| 408 | 41 | 2 | 412 | 0.5244 | 0.77103 | 1.967 | 1.3263 | 1.7919 | 2.46 |
| 409 | 41 | 2 | 412 | 0.478 | 0.73946 | 1.9122 | 0.7257 | 2.0611 | 3.128 |
| 410 | 41 | 2 | 412 | 0.1895 | 0.38713 | 1.3136 | 0.6262 | 2.1536 | 2.642 |
| 411 | 42 | 1 | 421 | 0.2141 | 0.46812 | 1.9988 | 0.7213 | 3.183 | 3.256 |
| 412 | 42 | 1 | 421 | 0.3112 | 0.47899 | 2.6068 | 1.2692 | 3.117 | 2.944 |
| 413 | 42 | 1 | 421 | 0.1948 | 0.87532 | 2.7126 | 1.3063 | 3.0311 | 3.078 |
| 414 | 42 | 1 | 421 | 0.2441 | 0.41304 | 2.3517 | 1.1109 | 3.2089 | 2.71 |
| 415 | 42 | 1 | 421 | 0.2886 | 0.4114 | 2.1141 | 1.5791 | 2.9766 | 3.09 |
| 416 | 42 | 2 | 422 | 0.1162 | 0.27713 | 1.0978 | 2.2511 | 1.0983 | 3.1 |
| 417 | 42 | 2 | 422 | 0.1599 | 0.39298 | 1.573 | 2.1579 | 0.9628 | 3.178 |
| 418 | 42 | 2 | 422 | 0.1167 | 0.30887 | 1.2591 | 1.5278 | 1.2919 | 2.198 |
| 419 | 42 | 2 | 422 | 0.2422 | 0.40353 | 2.1286 | 2.1536 | 1.0651 | 2.804 |
| 420 | 42 | 2 | 422 | 0.0579 | 0.43434 | 1.9089 | 1.6665 | 1.6477 | 2.968 |
| 421 | 43 | 1 | 431 | 0.3266 | 0.478 | 1.5533 | 1.7544 | 3.0683 | 2.982 |
| 422 | 43 | 1 | 431 | 0.2929 | 0.28322 | 1.0854 | 1.4507 | 2.3281 | 1.98 |
| 423 | 43 | 1 | 431 | 0.277 | 0.674 | 1.75 | 2.073 | 3.1188 | 2.788 |
| 424 | 43 | 1 | 431 | 0.2867 | 0.37058 | 1.8223 | 1.5258 | 2.3548 | 3.338 |
| 425 | 43 | 1 | 431 | 0.3058 | 0.50669 | 1.5322 | 2.3673 | 1.2612 | NA |
| 426 | 43 | 2 | 432 | 0.1297 | 0.15557 | 1.4046 | 1.2087 | 1.8414 | 2.08 |
| 427 | 43 | 2 | 432 | 0.1006 | 0.21385 | 1.3342 | 1.1639 | 1.5992 | 1.894 |
| 428 | 43 | 2 | 432 | 0.2055 | 0.33413 | 1.686 | 1.0099 | 1.5545 | 2.524 |
| 429 | 43 | 2 | 432 | 0.1552 | 0.28764 | 2.5052 | 1.2366 | 1.8843 | 2.774 |
| 430 | 43 | 2 | 432 | 0.1652 | 0.23425 | 1.6612 | 1.2425 | 1.4981 | 2.296 |
| 431 | 44 | 1 | 441 | 0.5322 | 0.92545 | 2.1688 | 2.0615 | 3.1286 | 2.732 |
| 432 | 44 | 1 | 441 | 0.2387 | 0.51437 | 1.9583 | 1.5065 | 1.9652 | 2.512 |
| 433 | 44 | 1 | 441 | 0.1738 | 0.31851 | 1.5908 | 1.1044 | 2.5586 | 2.404 |
| 434 | 44 | 1 | 441 | 0.2002 | 0.25444 | 1.0563 | 1.4533 | 2.2907 | 2.758 |
| 435 | 44 | 1 | 441 | 0.2124 | 0.38791 | 1.7896 | 1.2416 | 1.995 | 1.208 |
| 436 | 44 | 2 | 442 | 0.1886 | 0.47177 | 1.0009 | 0.5808 | 1.3847 | 2.124 |
| 437 | 44 | 2 | 442 | 0.1371 | 0.45996 | 1.2056 | 0.9856 | 1.0204 | 2.23 |
| 438 | 44 | 2 | 442 | 0.2174 | 0.51102 | 1.2704 | 1.0053 | 1.6696 | 2.458 |
| 439 | 44 | 2 | 442 | 0.2404 | 0.84662 | 1.5793 | 1.1298 | 1.3797 | 3.014 |
| 440 | 44 | 2 | 442 | 0.1522 | 0.44789 | 1.354 | 1.0866 | 1.5891 | 2.78 |
| 441 | 45 | 1 | 451 | 0.2169 | 0.53679 | 1.8982 | 1.3304 | 2.3941 | 3.05 |
| 442 | 45 | 1 | 451 | 0.2232 | 0.43383 | 1.9304 | 1.5763 | 2.7409 | 3.544 |
| 443 | 45 | 1 | 451 | 0.1725 | 0.37709 | 1.4984 | 1.1845 | 2.4751 | 2.99 |
| 444 | 45 | 1 | 451 | 0.1927 | 0.31253 | 1.3206 | 1.2534 | 3.2986 | 3.228 |
| 445 | 45 | 1 | 451 | 0.1195 | 0.19765 | 1.3472 | 1.0293 | 2.5654 | 3.044 |
| 446 | 45 | 2 | 452 | 0.22 | 0.60764 | 1.5256 | 2.1056 | 2.0137 | 2.886 |
| 447 | 45 | 2 | 452 | 0.1385 | 0.58215 | 1.8378 | 2.0584 | 1.6524 | 2.694 |
| 448 | 45 | 2 | 452 | 0.1438 | 0.374 | 2.1294 | 1.9118 | 1.5748 | 2.952 |
| 449 | 45 | 2 | 452 | 0.1338 | 0.25486 | 1.4181 | 2.1249 | 1.3016 | 3.406 |
| 450 | 45 | 2 | 452 | 0.0853 | 0.12821 | 1.023 | 1.3064 | 1.208 | 2.64 |
| 451 | 46 | 1 | 461 | 0.0929 | 0.18127 | 1.3879 | 0.9944 | 1.8165 | 2.534 |
| 452 | 46 | 1 | 461 | 0.2008 | 0.27387 | 1.5877 | 1.3087 | 2.8801 | 3.268 |
| 453 | 46 | 1 | 461 | 0.3393 | 0.36537 | 1.6134 | 1.6849 | 2.1914 | 2.808 |
| 454 | 46 | 1 | 461 | 0.1411 | 0.25176 | 0.8154 | 1.0301 | 1.9143 | 2.568 |
| 455 | 46 | 1 | 461 | 0.2063 | 0.37953 | 0.9089 | 0.973 | 2.2193 | 3.124 |
| 456 | 46 | 2 | 462 | 0.1654 | 0.42173 | 1.3021 | 1.5297 | 1.5079 | 2.776 |
| 457 | 46 | 2 | 462 | 0.1604 | 0.50668 | 1.8425 | 1.8757 | 2.0548 | 3.13 |
| 458 | 46 | 2 | 462 | 0.0813 | 0.34876 | 1.2541 | 1.5446 | 1.3832 | 2.51 |
| 459 | 46 | 2 | 462 | 0.105 | 0.64085 | 1.6621 | 1.885 | 1.8956 | 3.576 |
| 460 | 46 | 2 | 462 | 0.1306 | 0.44042 | 1.277 | 1.7313 | 1.7326 | 2.822 |
| 461 | 47 | 1 | 471 | 0.4636 | 0.54925 | 1.7375 | 1.0936 | 2.6594 | 4.452 |
| 462 | 47 | 1 | 471 | 0.0685 | 0.30088 | 1.9679 | 1.3136 | 3.0322 | 3.788 |
| 463 | 47 | 1 | 471 | 0.0891 | 0.24296 | 0.9859 | 0.9705 | 2.0134 | 3.268 |
| 464 | 47 | 1 | 471 | 0.0811 | 0.33195 | 0.9596 | 0.7176 | 2.1655 | 3.25 |
| 465 | 47 | 1 | 471 | 0.0712 | 0.16566 | 1.0126 | 0.8486 | 2.2091 | 2.732 |
| 466 | 47 | 2 | 472 | 0.1169 | 0.69212 | 1.9766 | 0.5646 | 2.5683 | NA |
| 467 | 47 | 2 | 472 | 0.1344 | 0.59207 | 1.7157 | 0.9072 | 2.9502 | 1.924 |
| 468 | 47 | 2 | 472 | 0.2485 | 0.78269 | 2.6196 | 1.4223 | 2.691 | 2.04 |
| 469 | 47 | 2 | 472 | 0.2368 | 0.86939 | 2.5955 | 1.1958 | 2.6609 | 2.328 |
| 470 | 47 | 2 | 472 | 0.2663 | 0.91949 | 2.3186 | 2.1535 | 1.546 | 2.122 |
| 471 | 48 | 1 | 481 | 0.0405 | 0.19376 | 1.1659 | 1.1177 | NA | NA |
| 472 | 48 | 1 | 481 | 0.1262 | 0.94178 | 1.4425 | 1.3464 | 3.073 | 3.258 |
| 473 | 48 | 1 | 481 | 0.144 | 0.74539 | 1.698 | 1.0834 | 3.1127 | 3.594 |
| 474 | 48 | 1 | 481 | 0.0546 | 0.59688 | 1.5583 | 0.7039 | 2.6584 | 3.308 |
| 475 | 48 | 1 | 481 | 0.181 | 0.66564 | 2.1126 | 1.2951 | 2.986 | 3.208 |
| 476 | 48 | 2 | 482 | 0.1333 | 0.88192 | 0.7402 | 3.0376 | 2.7473 | 2.96 |
| 477 | 48 | 2 | 482 | 0.124 | 0.61519 | 0.8886 | 3.4305 | 2.0136 | 3.282 |
| 478 | 48 | 2 | 482 | 0.1364 | 0.90052 | 0.7288 | 2.8931 | 2.6238 | 3.652 |
| 479 | 48 | 2 | 482 | 0.1689 | 0.77186 | 1.0146 | 2.8128 | 1.9616 | 2.736 |
| 480 | 48 | 2 | 482 | 0.0712 | 0.70567 | 0.7512 | 2.2195 | 1.8905 | 3.352 |
| 481 | 49 | 1 | 491 | 0.1442 | 0.66064 | 1.7262 | 1.9759 | 2.611 | 2.55 |
| 482 | 49 | 1 | 491 | 0.3506 | 0.76558 | 2.1025 | 1.9706 | 2.2167 | 3.178 |
| 483 | 49 | 1 | 491 | 0.2136 | 0.51001 | 1.8481 | 1.5494 | 2.2034 | 2.494 |
| 484 | 49 | 1 | 491 | 0.2708 | 0.92945 | 1.6143 | 2.1081 | 2.1177 | 3.23 |
| 485 | 49 | 1 | 491 | 0.3303 | 0.98872 | 1.9694 | 1.8195 | 1.7589 | 2.668 |
| 486 | 49 | 2 | 492 | 0.3572 | 0.48048 | 1.7335 | 0.3188 | 1.2791 | 1.582 |
| 487 | 49 | 2 | 492 | 0.3487 | 0.33537 | 1.4245 | 0.6791 | 1.0131 | 1.422 |
| 488 | 49 | 2 | 492 | 0.3095 | 0.41758 | 1.7628 | 0.6407 | 1.0644 | 0.67 |
| 489 | 49 | 2 | 492 | 0.2439 | 0.39468 | 1.5752 | 0.4416 | 1.2603 | 1.932 |
| 490 | 49 | 2 | 492 | 0.2173 | 0.35369 | 1.03649 | 0.5505 | 0.7234 | 2.08 |
| 491 | 50 | 1 | 501 | 0.1213 | 0.66119 | 1.6361 | 1.5572 | 2.3683 | 2.53 |
| 492 | 50 | 1 | 501 | 0.1115 | 0.8165 | 1.8705 | 1.9929 | 2.4087 | 2.754 |
| 493 | 50 | 1 | 501 | 0.2501 | 0.46687 | 1.8721 | 1.1136 | 1.8593 | 2.766 |
| 494 | 50 | 1 | 501 | 0.141 | 0.80413 | 1.6314 | 1.3816 | 1.8431 | 2.418 |
| 495 | 50 | 1 | 501 | 0.2506 | 0.74713 | 1.4639 | 1.1662 | 1.7576 | 2.462 |
| 496 | 50 | 2 | 502 | 0.1096 | 0.28866 | 0.5679 | 1.0059 | 1.6967 | 3.05 |
| 497 | 50 | 2 | 502 | 0.117 | 0.4363 | 0.9575 | 1.7168 | 1.039 | 2.138 |
| 498 | 50 | 2 | 502 | 0.1283 | 0.38513 | 0.5205 | 1.4897 | 1.0966 | 1.886 |
| 499 | 50 | 2 | 502 | 0.1684 | 0.28803 | 0.6489 | 1.599 | 0.5216 | 2.4 |
| 500 | 50 | 2 | 502 | 0.1467 | 0.64668 | 1.0552 | 1.9077 | 0.2536 | 1.122 |
| 501 | 51 | 1 | 511 | 0.1643 | 0.39192 | 1.3779 | 0.8868 | 1.2568 | 1.652 |
| 502 | 51 | 1 | 511 | 0.1472 | 0.2647 | 1.0521 | 1.002 | 1.7832 | 1.584 |
| 503 | 51 | 1 | 511 | 0.0955 | 0.41899 | 1.2274 | 1.1712 | 1.7964 | 1.612 |
| 504 | 51 | 1 | 511 | 0.0566 | 0.22424 | 1.379 | 0.8063 | 2.0059 | 1.712 |
| 505 | 51 | 1 | 511 | 0.1247 | 0.3406 | 1.6519 | 0.8995 | 1.9473 | 2.746 |
| 506 | 51 | 2 | 512 | 0.0812 | 0.55017 | 2.072 | 1.296 | 1.4852 | 2.982 |
| 507 | 51 | 2 | 512 | 0.1178 | 0.42237 | 2.5416 | 2.1936 | 1.5294 | 2.574 |
| 508 | 51 | 2 | 512 | 0.105 | 0.54438 | 1.8691 | 1.7489 | 1.4154 | 2.108 |
| 509 | 51 | 2 | 512 | 0.0932 | 0.73674 | 2.2212 | 1.9517 | 1.5348 | 2.734 |
| 510 | 51 | 2 | 512 | 0.1309 | 0.18252 | 1.5098 | 1.8509 | 1.0447 | 1.924 |
| 511 | 52 | 1 | 521 | 0.0924 | 0.51473 | 1.1986 | 1.3919 | 1.5816 | 3.002 |
| 512 | 52 | 1 | 521 | 0.1221 | 0.65884 | 1.2073 | 1.1633 | 1.5954 | 2.848 |
| 513 | 52 | 1 | 521 | 0.1278 | 0.65645 | 1.5002 | 1.5544 | 1.8822 | 3.586 |
| 514 | 52 | 1 | 521 | 0.0628 | 0.49765 | 1.9654 | 2.0725 | 1.8921 | 3.236 |
| 515 | 52 | 1 | 521 | 0.046 | 0.39861 | 0.9953 | 1.8504 | 2.1151 | 2.474 |
| 516 | 52 | 2 | 522 | 0.1256 | 0.5809 | 1.7403 | 1.4841 | 1.5361 | 2.206 |
| 517 | 52 | 2 | 522 | 0.1442 | 0.5872 | 1.2659 | 0.914 | 1.3233 | 2.084 |
| 518 | 52 | 2 | 522 | 0.1012 | 0.4878 | 1.2189 | 0.9563 | 1.6081 | 2.704 |
| 519 | 52 | 2 | 522 | 0.117 | 0.5849 | 1.3048 | 1.1427 | 1.3909 | 1.812 |
| 520 | 52 | 2 | 522 | 0.137 | 0.4571 | 1.4011 | 1.3876 | 1.571 | 2.288 |
| 521 | 53 | 1 | 531 | 0.1047 | 0.54863 | 1.4715 | 2.0072 | 1.8222 | 3.26 |
| 522 | 53 | 1 | 531 | 0.1295 | 0.52548 | 1.5415 | 1.7062 | 1.9963 | 3.384 |
| 523 | 53 | 1 | 531 | 0.0776 | 0.42254 | 1.1351 | 2.2879 | 1.9718 | 3.93 |
| 524 | 53 | 1 | 531 | 0.3896 | 0.48085 | 1.4362 | 1.4132 | 2.3704 | 3.108 |
| 525 | 53 | 1 | 531 | 0.2622 | 0.58907 | 2.0254 | 1.4875 | 2.2713 | 3.666 |
| 526 | 53 | 2 | 532 | 0.181 | 0.1843 | 2.2154 | 1.5842 | 2.5735 | 2.996 |
| 527 | 53 | 2 | 532 | 0.2172 | 0.43745 | 2.0685 | 1.7045 | 1.665 | 3.716 |
| 528 | 53 | 2 | 532 | 0.183 | 0.34759 | 1.406 | 1.2937 | 0.998 | 3.328 |
| 529 | 53 | 2 | 532 | 0.134 | 0.24129 | 1.53 | 1.1435 | 1.6648 | 3.78 |
| 530 | 53 | 2 | 532 | 0.1597 | 0.3625 | 1.6363 | 0.8938 | 1.442 | 3.776 |
| 531 | 54 | 1 | 541 | 0.1495 | 0.35855 | 1.2337 | 0.9651 | 2.3952 | 3.772 |
| 532 | 54 | 1 | 541 | 0.3125 | 0.83163 | 2.1597 | 2.1561 | 2.941 | 2.93 |
| 533 | 54 | 1 | 541 | 0.3488 | 0.35639 | 1.5074 | 1.3929 | 2.2606 | 3.53 |
| 534 | 54 | 1 | 541 | 0.2379 | 0.43993 | 2.1555 | 1.6577 | 2.7561 | 4.132 |
| 535 | 54 | 1 | 541 | 0.3304 | 0.20061 | 1.622 | 1.3405 | 1.9751 | 2.844 |
| 536 | 54 | 2 | 542 | 0.1911 | 0.80107 | 2.2223 | 1.2546 | 2.4146 | 2.97 |
| 537 | 54 | 2 | 542 | 0.1573 | 0.42363 | 1.9135 | 0.8892 | 2.5584 | 2.83 |
| 538 | 54 | 2 | 542 | 0.2581 | 0.64186 | 2.2077 | 1.5944 | 2.3465 | 2.76 |
| 539 | 54 | 2 | 542 | 0.2226 | 0.49534 | 1.7528 | 1.5005 | 2.5373 | 3.82 |
| 540 | 54 | 2 | 542 | 0.2282 | 0.80311 | 1.5739 | 2.3343 | 2.1719 | 3.25 |
| 541 | 55 | 1 | 551 | 0.5387 | 0.30404 | 1.4095 | 1.2973 | 0.8903 | 2.274 |
| 542 | 55 | 1 | 551 | 0.6056 | 0.25345 | 1.9325 | 1.1119 | 1.9918 | 3.212 |
| 543 | 55 | 1 | 551 | 0.406 | 0.21511 | 1.424 | 0.7111 | 1.4896 | 1.99 |
| 544 | 55 | 1 | 551 | 0.2766 | 0.3282 | 1.4572 | 0.9748 | 1.4504 | 2.074 |
| 545 | 55 | 1 | 551 | 0.434 | 0.2561 | 1.6178 | 1.1038 | 2.2089 | 3.27 |
| 546 | 55 | 2 | 552 | 0.1629 | 0.53067 | 1.3993 | 1.3382 | 2.1631 | NA |
| 547 | 55 | 2 | 552 | 0.1707 | 0.47806 | 1.2671 | 0.7016 | 1.2339 | NA |
| 548 | 55 | 2 | 552 | 0.1956 | 0.62794 | 1.1372 | 1.2704 | 1.3731 | NA |
| 549 | 55 | 2 | 552 | 0.2398 | 0.60358 | 1.1121 | 1.1953 | 1.2776 | NA |
| 550 | 55 | 2 | 552 | 0.2193 | 0.60026 | 1.8502 | 1.1314 | 1.4297 | NA |
| 551 | 56 | 1 | 561 | 0.333 | 0.21086 | 1.3131 | 1.7992 | 2.7776 | 3.34 |
| 552 | 56 | 1 | 561 | 0.3433 | 0.40035 | 1.2279 | 2.0103 | 3.2061 | 3.004 |
| 553 | 56 | 1 | 561 | 0.2839 | 0.316 | 1.4822 | 2.0816 | 3.4218 | 3.968 |
| 554 | 56 | 1 | 561 | 0.164 | 0.22981 | 1.3656 | 2.2527 | 3.0866 | 3.578 |
| 555 | 56 | 1 | 561 | 0.1057 | 0.38806 | 1.3246 | 2.1575 | 2.4772 | 3.388 |
| 556 | 56 | 2 | 562 | 0.1748 | 0.4914 | 2.3867 | 2.3563 | 2.4364 | 2.764 |
| 557 | 56 | 2 | 562 | 0.0935 | 0.2378 | 1.6603 | 1.9342 | 1.9998 | 3.19 |
| 558 | 56 | 2 | 562 | 0.1381 | 0.4098 | 1.9306 | 1.8875 | 2.2067 | 2.162 |
| 559 | 56 | 2 | 562 | 0.0473 | 0.591 | 1.242 | 1.0964 | 2.545 | 2.332 |
| 560 | 56 | 2 | 562 | 0.1983 | 0.5138 | 1.9203 | 1.9143 | 2.265 | 2.644 |
| 561 | 57 | 1 | 571 | 0.0367 | 0.47316 | 1.8142 | 0.772 | 1.2951 | 5.01 |
| 562 | 57 | 1 | 571 | 0.125 | 0.21588 | 0.4012 | 0.1292 | 0.4705 | 1.608 |
| 563 | 57 | 1 | 571 | 0.0782 | 0.08086 | 0.3059 | 0.1716 | 0.3655 | 1.35 |
| 564 | 57 | 1 | 571 | 0.083 | 0.84563 | 0.3627 | 0.5244 | 0.527 | 1.618 |
| 565 | 57 | 1 | 571 | 0.0822 | 0.08791 | 0.2502 | 0.1946 | 0.3302 | 1.55 |
| 566 | 57 | 2 | 572 | NA | NA | NA | NA | NA | NA |
| 567 | 57 | 2 | 572 | NA | NA | NA | NA | NA | NA |
| 568 | 57 | 2 | 572 | NA | NA | NA | NA | NA | NA |
| 569 | 57 | 2 | 572 | NA | NA | NA | NA | NA | NA |
| 570 | 57 | 2 | 572 | NA | NA | NA | NA | NA | NA |
| 571 | 58 | 1 | 581 | 0.2347 | 0.53066 | 0.753 | 1.8928 | 1.762 | 3.64 |
| 572 | 58 | 1 | 581 | 0.2062 | 0.29052 | 0.825 | 1.4644 | 2.5939 | 3.838 |
| 573 | 58 | 1 | 581 | 0.2144 | 0.39564 | 0.7933 | 2.2168 | 1.6583 | 3.43 |
| 574 | 58 | 1 | 581 | 0.1636 | 0.44352 | 0.8632 | 1.8136 | 1.8793 | 3.388 |
| 575 | 58 | 1 | 581 | 0.1221 | 0.40452 | 0.7431 | 2.2916 | 2.0495 | 3.008 |
| 576 | 58 | 2 | 582 | 0.2785 | 0.42997 | 1.1295 | 0.9147 | 1.3569 | 1.946 |
| 577 | 58 | 2 | 582 | 0.3358 | 0.58052 | 1.7612 | 1.22 | 2.3391 | 2.71 |
| 578 | 58 | 2 | 582 | 0.2395 | 0.56827 | 1.7733 | 1.4632 | 2.2831 | 2.76 |
| 579 | 58 | 2 | 582 | 0.363 | 0.4457 | 2.0675 | 0.846 | 2.8848 | 2.922 |
| 580 | 58 | 2 | 582 | 0.228 | 0.20789 | 1.4079 | 0.4657 | 1.7684 | 2.308 |
| 581 | 59 | 1 | 591 | 0.1621 | 0.33922 | 1.145 | 0.8841 | 2.153 | 4.776 |
| 582 | 59 | 1 | 591 | 0.2946 | 0.31922 | 1.0379 | 0.6777 | 2.1047 | 4.89 |
| 583 | 59 | 1 | 591 | 0.1821 | 0.29114 | 1.0565 | 0.8926 | 1.6806 | 3.22 |
| 584 | 59 | 1 | 591 | 0.107 | 0.51084 | 1.5809 | 0.9917 | 2.5336 | 3.98 |
| 585 | 59 | 1 | 591 | 0.2186 | 0.32277 | 1.6258 | 1.3485 | 2.6716 | 3.78 |
| 586 | 59 | 2 | 592 | 0.0495 | 0.43679 | 0.3369 | 0.5425 | 1.0009 | 2.418 |
| 587 | 59 | 2 | 592 | 0.0554 | 0.27625 | 0.1485 | 0.3403 | 1.5331 | 2.59 |
| 588 | 59 | 2 | 592 | 0.1021 | 0.31826 | 0.2131 | 0.2671 | 1.6241 | 3.13 |
| 589 | 59 | 2 | 592 | 0.0254 | 0.35067 | 0.3613 | 0.1855 | 1.0254 | 1.864 |
| 590 | 59 | 2 | 592 | 0.0546 | 0.31235 | 0.1968 | 0.3572 | 1.0577 | 2.574 |
| 591 | 60 | 1 | 601 | 0.0705 | 0.16559 | 0.7927 | 0.989 | 1.5058 | 2.438 |
| 592 | 60 | 1 | 601 | 0.2442 | 0.46024 | 1.5151 | 1.1864 | 1.6445 | 2.62 |
| 593 | 60 | 1 | 601 | 0.1888 | 0.36207 | 0.9887 | 0.9374 | 1.1529 | 2.19 |
| 594 | 60 | 1 | 601 | 0.2295 | 0.2568 | 1.6237 | 1.0402 | 1.2285 | 2.108 |
| 595 | 60 | 1 | 601 | 0.3928 | 0.32513 | 1.7453 | 1.6018 | 1.8086 | 2.376 |
| 596 | 60 | 2 | 602 | 0.1191 | 0.34573 | 1.2047 | 1.2872 | 1.3371 | 2.814 |
| 597 | 60 | 2 | 602 | 0.132 | 0.26291 | 1.5174 | 1.74816 | 1.2525 | 3.844 |
| 598 | 60 | 2 | 602 | 0.1604 | 0.24969 | 1.8758 | 2.02228 | 1.8076 | 3.62 |
| 599 | 60 | 2 | 602 | 0.2083 | 0.63967 | 2.568 | 2.57006 | 2.1773 | 3.86 |
| 600 | 60 | 2 | 602 | 0.1453 | 0.26854 | 1.3537 | 2.5668 | 1.9815 | 3.508 |
| 601 | 61 | 1 | 611 | 0.0941 | 0.48459 | 1.9021 | 1.4743 | 1.4218 | 3.316 |
| 602 | 61 | 1 | 611 | 0.1482 | 0.55593 | 1.624 | 1.3346 | 1.3412 | 3.238 |
| 603 | 61 | 1 | 611 | 0.2085 | 0.89914 | 2.2 | 2.0399 | 1.6625 | 3.58 |
| 604 | 61 | 1 | 611 | 0.2251 | 0.4412 | 2.0144 | 1.6523 | 1.9558 | 3.37 |
| 605 | 61 | 1 | 611 | 0.1445 | 0.58096 | 2.4288 | 1.1672 | 1.7157 | 3.508 |
| 606 | 61 | 2 | 612 | 0.0699 | 0.63453 | 1.5702 | 1.6125 | 2.1908 | 2.892 |
| 607 | 61 | 2 | 612 | 0.2588 | 0.87144 | 2.5087 | 1.94475 | 2.2904 | 3.432 |
| 608 | 61 | 2 | 612 | 0.1009 | 0.45301 | 0.9564 | 1.80879 | 1.2944 | 2.76 |
| 609 | 61 | 2 | 612 | 0.1381 | 0.41344 | 1.365 | 2.08414 | 1.4631 | 3.624 |
| 610 | 61 | 2 | 612 | 0.2283 | 0.4014 | 1.5333 | 1.67231 | 1.5664 | 3.884 |
| 611 | 62 | 1 | 621 | 0.1926 | 0.82877 | 2.6233 | 1.3127 | 2.5446 | 3.388 |
| 612 | 62 | 1 | 621 | 0.1156 | 0.95801 | 2.1513 | 1.7258 | 1.6446 | 3.82 |
| 613 | 62 | 1 | 621 | 0.1324 | 0.69503 | 1.9639 | 1.4629 | 2.0577 | 3.102 |
| 614 | 62 | 1 | 621 | 0.1507 | 0.54313 | 2.0763 | 1.2963 | 2.0017 | 3.082 |
| 615 | 62 | 1 | 621 | 0.1746 | 0.55603 | 2.3474 | 1.321 | 1.4085 | 4.02 |
| 616 | 62 | 2 | 622 | 0.1218 | 0.72265 | 1.7515 | 1.6671 | 2.239 | 3.036 |
| 617 | 62 | 2 | 622 | 0.2036 | 0.39693 | 1.5986 | 1.9885 | 1.6623 | 3.684 |
| 618 | 62 | 2 | 622 | 0.2108 | 0.6463 | 1.6635 | 1.2476 | 1.7808 | 3.184 |
| 619 | 62 | 2 | 622 | 0.1986 | 0.42346 | 1.1894 | 0.7802 | 1.2478 | 2.822 |
| 620 | 62 | 2 | 622 | 0.2124 | 0.47476 | 1.4418 | 1.0262 | 1.4176 | 2.658 |
| 621 | 63 | 1 | 631 | 0.1867 | 0.51295 | 1.7288 | 1.5017 | 1.6151 | 4.38 |
| 622 | 63 | 1 | 631 | 0.1261 | 0.54729 | 1.5194 | 1.26553 | 1.8592 | 4.02 |
| 623 | 63 | 1 | 631 | 0.2031 | 0.5359 | 1.1841 | 1.3601 | 1.6494 | 3.315 |
| 624 | 63 | 1 | 631 | 0.1934 | 1.03626 | 2.8362 | 1.96666 | 1.9916 | 4.035 |
| 625 | 63 | 1 | 631 | 0.0613 | 0.44901 | 1.3964 | 1.34419 | 1.4933 | 3.43 |
| 626 | 63 | 2 | 632 | 0.09 | 0.20978 | 0.6607 | 0.6825 | 0.9242 | 2.14 |
| 627 | 63 | 2 | 632 | 0.0859 | 0.26767 | 0.485 | 0.3763 | 1.2256 | 2.484 |
| 628 | 63 | 2 | 632 | 0.0401 | 0.25264 | 0.9154 | 0.3068 | 1.2851 | 2.398 |
| 629 | 63 | 2 | 632 | 0.02495 | 0.33322 | 1.0171 | 0.6392 | 1.0514 | 2.33 |
| 630 | 63 | 2 | 632 | 0.0568 | 0.35112 | 1.221 | 0.6415 | 1.0996 | 2.694 |
| 631 | 64 | 1 | 641 | 0.2773 | 0.59128 | 1.6955 | 1.1372 | 2.2772 | 1.865 |
| 632 | 64 | 1 | 641 | 0.1625 | 0.2816 | 1.2034 | 0.5281 | 1.0243 | 2.11 |
| 633 | 64 | 1 | 641 | 0.2149 | 0.42353 | 1.4671 | 0.9796 | 1.5506 | 2.97 |
| 634 | 64 | 1 | 641 | 0.1863 | 0.32816 | 1.4634 | 0.6622 | 1.252 | 2.39 |
| 635 | 64 | 1 | 641 | 0.1093 | 0.28737 | 1.9797 | 0.5377 | 0.8406 | 2.905 |
| 636 | 64 | 2 | 642 | 0.0843 | 0.78776 | 2.1424 | 2.6407 | 0.8389 | 3.252 |
| 637 | 64 | 2 | 642 | 0.2937 | 0.62322 | 2.0944 | 2.9681 | 0.8502 | 3.862 |
| 638 | 64 | 2 | 642 | 0.1674 | 0.72403 | 2.4405 | 2.9219 | 1.4337 | 3.814 |
| 639 | 64 | 2 | 642 | 0.1655 | 0.33689 | 1.2375 | 2.4364 | 1.297 | 3.596 |
| 640 | 64 | 2 | 642 | 0.346 | 0.54873 | 1.8533 | 2.3488 | 1.382 | 4.318 |
| 641 | 65 | 1 | 651 | 0.3038 | 0.42516 | 2.6104 | 0.9823 | 2.0259 | 3.71 |
| 642 | 65 | 1 | 651 | 0.3657 | 0.39757 | 2.285 | 1.3537 | 1.508 | 2.962 |
| 643 | 65 | 1 | 651 | 0.2713 | 0.35511 | 1.5914 | 1.0765 | 1.1005 | 2.756 |
| 644 | 65 | 1 | 651 | 0.4333 | 0.61008 | 1.9594 | 0.7161 | 1.382 | 2.426 |
| 645 | 65 | 1 | 651 | 0.3121 | 0.42927 | 1.8611 | 1.2047 | 1.4082 | 3.646 |
| 646 | 65 | 2 | 652 | 0.1654 | 0.92833 | 2.2374 | 0.3095 | 2.348 | 4.026 |
| 647 | 65 | 2 | 652 | 0.1554 | 1.05187 | 2.7052 | 1.86974 | 2.1163 | 3.226 |
| 648 | 65 | 2 | 652 | 0.1472 | 0.49536 | 1.4876 | 1.71254 | 1.8247 | 3.658 |
| 649 | 65 | 2 | 652 | 0.1414 | 0.61136 | 1.4969 | 1.38711 | 2.6179 | 3.18 |
| 650 | 65 | 2 | 652 | 0.1628 | 0.50451 | 1.9162 | 0.78327 | 2.651 | 3.116 |
| 651 | 66 | 1 | 661 | 0.3159 | 0.35431 | 1.0913 | 1.75836 | 2.168 | 3.384 |
| 652 | 66 | 1 | 661 | 0.1962 | 0.26955 | 1.1863 | 0.98934 | 1.7941 | 3.482 |
| 653 | 66 | 1 | 661 | 0.1688 | 0.32693 | 0.8361 | 1.13527 | 1.0609 | 3.104 |
| 654 | 66 | 1 | 661 | 0.2261 | 0.7562 | 3.1056 | 1.64627 | 2.0802 | 3.786 |
| 655 | 66 | 1 | 661 | 0.1194 | 0.25976 | 1.3641 | 0.92212 | 1.8085 | 2.864 |
| 656 | 66 | 2 | 662 | 0.0495 | 0.10911 | 0.1712 | 1.0574 | 1.3221 | 2.396 |
| 657 | 66 | 2 | 662 | 0.0426 | 0.10131 | 0.133 | 1.1038 | 0.8088 | 1.686 |
| 658 | 66 | 2 | 662 | 0.0532 | 0.23276 | 0.0293 | 0.8094 | 0.5704 | 1.562 |
| 659 | 66 | 2 | 662 | 0.0754 | 0.12807 | 0.0806 | 0.4449 | 0.8592 | 1.216 |
| 660 | 66 | 2 | 662 | 0.0422 | 0.25964 | 0.0894 | 0.263 | 0.8411 | 1.6 |
| 661 | 67 | 1 | 671 | 0.1797 | 0.26104 | 2.7819 | 2.978 | 2.6899 | 3.584 |
| 662 | 67 | 1 | 671 | 0.1919 | 0.39851 | 2.4982 | 2.2237 | 2.015 | 3.856 |
| 663 | 67 | 1 | 671 | 0.3761 | 0.44005 | 1.5123 | 2.125 | 1.7573 | 2.796 |
| 664 | 67 | 1 | 671 | 0.5352 | 0.53534 | 2.2652 | 2.1937 | 1.9806 | 3.54 |
| 665 | 67 | 1 | 671 | 0.4501 | 0.29214 | 2.8532 | 2.3722 | 2.0184 | 3.636 |
| 666 | 67 | 2 | 672 | 0.1246 | 0.48181 | 1.0654 | 1.21142 | 2.4209 | 3.626 |
| 667 | 67 | 2 | 672 | 0.2434 | 0.58808 | 2.3551 | 1.65364 | 2.3364 | 3.996 |
| 668 | 67 | 2 | 672 | 0.1282 | 0.34161 | 1.4924 | 0.78962 | 1.1896 | 1.972 |
| 669 | 67 | 2 | 672 | 0.1162 | 0.28878 | 0.6235 | 1.73674 | 1.1439 | 2.45 |
| 670 | 67 | 2 | 672 | 0.1203 | 0.24279 | 2.0432 | 1.79963 | 1.8562 | 3.426 |
| 671 | 68 | 1 | 681 | 0.4179 | 0.71331 | 1.1533 | 3.0628 | 2.1971 | 4.01 |
| 672 | 68 | 1 | 681 | 0.3052 | 0.78755 | 1.4327 | 2.2762 | 2.3026 | 4.368 |
| 673 | 68 | 1 | 681 | 0.1662 | 1.04403 | 1.7577 | 2.5842 | 2.8687 | 4.51 |
| 674 | 68 | 1 | 681 | 0.1655 | 0.45473 | 1.3814 | 2.3686 | 2.0566 | 5.43 |
| 675 | 68 | 1 | 681 | 0.279 | NA | NA | NA | NA | NA |
| 676 | 68 | 2 | 682 | 0.2776 | 0.23654 | 1.7606 | 0.6852 | 1.1372 | 2.66 |
| 677 | 68 | 2 | 682 | 0.3411 | 0.30716 | 1.6626 | 1.1528 | 1.3214 | 2.14 |
| 678 | 68 | 2 | 682 | 0.2607 | 0.30819 | 1.3572 | 0.7627 | 1.3995 | 1.818 |
| 679 | 68 | 2 | 682 | 0.2722 | 0.53376 | 1.5731 | 1.3112 | 1.7619 | 1.734 |
| 680 | 68 | 2 | 682 | 0.1489 | 0.33516 | 0.9364 | 1.0644 | 1.8918 | 2.128 |
| 681 | 69 | 1 | 691 | 0.2763 | 0.43029 | 2.1371 | 2.6498 | 3.1669 | 4.578 |
| 682 | 69 | 1 | 691 | 0.1411 | 0.30027 | 1.2758 | 1.3449 | 1.8802 | 3.818 |
| 683 | 69 | 1 | 691 | 0.4235 | 0.26606 | 0.7797 | 0.5086 | 1.7434 | 3.752 |
| 684 | 69 | 1 | 691 | 0.1813 | 0.43292 | 0.7858 | 0.6517 | 1.3934 | 1.838 |
| 685 | 69 | 1 | 691 | 0.3125 | 0.40888 | 0.8303 | 0.8741 | 1.7763 | 2.04 |
| 686 | 69 | 2 | 692 | 0.1733 | 0.55078 | 1.9823 | 0.8094 | 2.5512 | 2.661 |
| 687 | 69 | 2 | 692 | 0.1168 | 0.3573 | 1.1537 | 0.7725 | 1.9715 | 2.608 |
| 688 | 69 | 2 | 692 | 0.1698 | 0.50412 | 1.7619 | 1.4435 | 2.3702 | 3.162 |
| 689 | 69 | 2 | 692 | 0.1431 | 0.38043 | 2.2151 | 1.4398 | 1.9955 | 2.002 |
| 690 | 69 | 2 | 692 | 0.2062 | 0.38923 | 2.2041 | 1.587 | 1.923 | 2.179 |
| 691 | 70 | 1 | 701 | 0.3444 | 0.73965 | 0.3147 | 2.5376 | 3.2985 | 3.572 |
| 692 | 70 | 1 | 701 | 0.1391 | 0.88775 | 0.5272 | 3.6428 | 3.045 | 4.408 |
| 693 | 70 | 1 | 701 | 0.2504 | 0.84202 | 0.3146 | 2.9479 | 2.9353 | 3.998 |
| 694 | 70 | 1 | 701 | 0.1064 | 0.64856 | 0.4182 | 2.7696 | 2.2231 | 3.578 |
| 695 | 70 | 1 | 701 | 0.2739 | 0.66217 | 0.4065 | 2.8571 | 3.4446 | 4.212 |
| 696 | 70 | 2 | 702 | 0.1708 | 0.86505 | 0.7327 | 1.0379 | 2.0981 | 2.482 |
| 697 | 70 | 2 | 702 | 0.1551 | 0.89722 | 0.8915 | 1.0886 | 1.8463 | 3.49 |
| 698 | 70 | 2 | 702 | 0.2676 | 1.06739 | 0.6389 | 1.117 | 1.8411 | 2.788 |
| 699 | 70 | 2 | 702 | 0.268 | 0.82676 | 1.1298 | 2.2941 | 2.6009 | 3.818 |
| 700 | 70 | 2 | 702 | NA | 0.48986 | NA | NA | 2.4697 | 3.052 |
| 701 | 71 | 1 | 711 | 0.3045 | 0.88657 | 1.5399 | 3.1425 | 3.7846 | 3.36 |
| 702 | 71 | 1 | 711 | 0.39 | 0.75706 | 1.6528 | 2.2073 | 2.6703 | 3.878 |
| 703 | 71 | 1 | 711 | 0.4651 | 0.7619 | 1.7645 | 1.9698 | 2.557 | 3.22 |
| 704 | 71 | 1 | 711 | 0.4272 | 0.72791 | 1.4198 | 1.2732 | 2.6612 | 2.722 |
| 705 | 71 | 1 | 711 | 0.261 | 0.35771 | 1.0633 | 0.9304 | 2.2837 | 2.88 |
| 706 | 71 | 2 | 712 | NA | 0.03311 | 0.1676 | NA | 0.5535 | 1.798 |
| 707 | 71 | 2 | 712 | 0.4401 | 0.84495 | 1.2081 | 1.7977 | 2.0761 | 4.23 |
| 708 | 71 | 2 | 712 | 0.319 | 0.65109 | 1.154 | 1.2657 | 0.5587 | 1.04 |
| 709 | 71 | 2 | 712 | 0.3493 | 0.70806 | 1.3318 | 1.1875 | 1.4451 | 2.939 |
| 710 | 71 | 2 | 712 | 0.2725 | 0.86028 | 1.773 | 1.5075 | 1.6533 | 3.004 |
| 711 | 72 | 1 | 721 | 0.4581 | 0.74728 | 1.8753 | 1.7874 | 2.5385 | 3.164 |
| 712 | 72 | 1 | 721 | 0.428 | 0.92721 | 1.5487 | 1.5023 | 3.8472 | 3.582 |
| 713 | 72 | 1 | 721 | 0.4952 | 0.48218 | 1.874 | 2.0264 | 3.016 | 3.848 |
| 714 | 72 | 1 | 721 | 0.2736 | 0.72065 | 1.8293 | 2.0521 | 2.9844 | 4.098 |
| 715 | 72 | 1 | 721 | 0.4924 | 0.94637 | 2.3211 | 1.6887 | 3.0206 | 3.404 |
| 716 | 72 | 2 | 722 | 0.2267 | 0.35305 | 2.2672 | 0.9369 | 2.0833 | 2.762 |
| 717 | 72 | 2 | 722 | 0.4687 | 0.27302 | 1.0713 | 0.5373 | 1.9933 | 3.096 |
| 718 | 72 | 2 | 722 | 0.3311 | 0.37506 | 0.9604 | 0.6888 | 1.5081 | 2.05 |
| 719 | 72 | 2 | 722 | 0.2815 | 0.41111 | 0.4717 | 0.4726 | 1.483 | 1.088 |
| 720 | 72 | 2 | 722 | 0.2141 | 0.20402 | 0.746 | 1.2684 | 1.3569 | 1.382 |
| 721 | 73 | 1 | 731 | 0.1097 | 0.58592 | 1.3579 | 2.2103 | 2.6634 | 3.248 |
| 722 | 73 | 1 | 731 | 0.0944 | 0.4462 | 1.1498 | 1.961 | 2.5222 | 3.498 |
| 723 | 73 | 1 | 731 | 0.1065 | 0.27112 | 0.638 | 2.133 | 2.7642 | 3.009 |
| 724 | 73 | 1 | 731 | 0.2879 | 0.44898 | 1.0228 | 3.0272 | 1.8929 | 3.808 |
| 725 | 73 | 1 | 731 | 0.1625 | 0.55978 | 1.5287 | 3.5613 | 3.6018 | 5.238 |
| 726 | 73 | 2 | 732 | 0.1552 | 0.44709 | 1.1053 | 1.7691 | 1.953 | 1.914 |
| 727 | 73 | 2 | 732 | 0.2518 | 0.51273 | 1.3324 | 1.5782 | 1.9958 | 2.93 |
| 728 | 73 | 2 | 732 | 0.3224 | 0.54662 | 1.636 | 1.2696 | 2.7155 | 2.784 |
| 729 | 73 | 2 | 732 | 0.1327 | 0.68455 | 1.8347 | 0.8133 | 2.1382 | 2.988 |
| 730 | 73 | 2 | 732 | 0.1228 | 0.36432 | 1.1922 | 1.0857 | 1.7189 | 3.67 |
